# Epithelial cell shape changes contribute to regulation of ureteric bud branching morphogenesis

**DOI:** 10.1111/febs.70156

**Published:** 2025-06-15

**Authors:** Kristen Kurtzeborn, Vladislav Iaroshenko, Tomáš Zárybnický, Julia Koivula, Heidi Anttonen, Otto J. M. Mäkelä, Darren Bridgewater, Ramaswamy Krishnan, Ping Chen, Satu Kuure

**Affiliations:** ^1^ Helsinki Institute of Life Science University of Helsinki Finland; ^2^ Stem Cells and Metabolism Research Program Unit, Faculty of Medicine University of Helsinki Finland; ^3^ Department of Biochemistry and Developmental Biology, Faculty of Medicine University of Helsinki Finland; ^4^ Department of Pathology and Molecular Medicine McMaster University Hamilton Canada; ^5^ Center for Vascular Biology Research, Department of Emergency Medicine Beth Israel Deaconess Medical Center Boston MA USA; ^6^ Division of Clinical Chemistry, Department of Laboratory Medicine Karolinska Institutet Stockholm Sweden; ^7^ Laboratory Animal Centre University of Helsinki Finland

**Keywords:** biomechanics, branching morphogenesis, cell sizes, kidney development, mechanical forces

## Abstract

Branching morphogenesis orchestrates organogenesis in many tissues, including the kidney, where ureteric bud (UB) branching determines kidney size and shape and the final nephron number. Molecular regulation of UB branching is rather well studied, whereas the cellular mechanisms and tissue organization during UB arborization are less understood. Here, we characterized epithelial cell morphology in three dimensions (3D), studied mechanisms regulating cell shape changes, and analyzed their contribution to novel branch initiation in normal and branching‐incompetent bud tips. Unbiased machine‐learning‐based segmentation of tip epithelia identified geometrical round‐to‐elliptical transformation as a key cellular mechanism facilitating growth direction changes to gain optimal branching complexity. Cell shape and molecular analyses in branching‐incompetent epithelia demonstrated a distinct failure to condense cell size and modify its conformation. This, together with changes in adhesive forces, defective actin dynamics, and disorganization in myosin‐9 (MYH9)‐based microtubules, suggests altered biophysical properties in tip cells, where branch point decisions are made and actualized. The data demonstrate that dynamic changes in volume and morphology of individual epithelial cells, together with optimal traction stress, facilitate novel branch formation in the UB tip niche. Based on these results, we propose a model where epithelial cell crowding, in tandem with stretching, transforms individual cells into elliptical and elongated shapes. This creates local curvatures that drive new branch formation during the ampulla‐to‐asymmetric ampulla transition of the UB.

## Graphical abstract

Ureteric bud (UB) branching determines the final size, shape, and nephron number of kidneys. Characterization of UB cell shapes identified dynamic round‐to‐elliptical transitions and cellular volume changes as critical epithelial modifications that increase morphological complexity in UB tips during their transitions through ampulla‐to‐asymmetric ampulla conformations. Our findings support the cellular crowding model in transforming individual cells into elliptical and elongated shapes to facilitate diversity in decisions of new branch orientations.

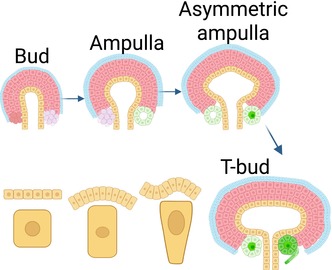

**Abbreviations**
3D: three dimensional
AI: artificial intelligence
BC: branching competent
BI: branching incompetent
DEG: differentially expressed gene
E‐CAD: E‐CADHERIN
EGTA: ethylene glycol tetraacetic acid
ERK: extracellular signal‐regulated kinase
GFP: green fluorescent protein
MAPK: mitogen‐activated protein kinase
MYH9: myosin, heavy polypeptide 9, non‐muscle
ppMLC: phosphorylated MYOSIN Light Chain
UB: ureteric bud
UMAP: uniform manifold approximation and projection

## Introduction

The development of many organs depends on complex epithelial tube formation known as branching morphogenesis [1, 2, 3]. Branching involves repeated creation of new branch points with simultaneous tube elongations to maximize functional area in a limited 3D space. Two main mechanisms of branching have been described: tip bifurcation (clefting) and side (lateral) branching [4, 5]. In the lung and kidney, stereotyped branching morphogenesis predominates, but the patterns differ from each other by the presence or absence of lateral branching events. In the lung, three geometrically distinct modes of branching exist: domain (lateral) branching, planar bifurcation, and orthogonal bifurcation, and each of these modes occurs in a fixed order [6]. In the kidney, the ureteric bud (UB) undergoes reiterative tip bifurcations early in development followed by some trifurcations during later development. The morphological changes in the UB tip give rise to an epithelial tree where the extent and gross pattern of branch elaboration are conserved and highly reproducible [7].

Extensive cell rearrangements and geometry changes sculpt epithelia in different developing organs [8]. Coordinated cell shape changes in epithelium bend the tissue leading to variations in morphological organization. Dynamic cellular adhesions coupled to tightly regulated cell shape changes contribute to tissue remodeling in developing organs of *Drosophila* [9, 10, 11]. Moreover, epithelial integrity and morphology in mature mammalian collecting ducts depend on the fine control of the actomyosin network dynamics involving actin polymerization, which was previously shown to be required for normal UB branching [12, 13]. Inhibition of myosin function in mouse lung resulted in abnormal cell shapes, suggesting functions in constraining cell morphology, which, however, must be released at the sites of new branch points [14]. The function of two non‐muscle myosin heavy chain proteins, MYH9 and MYH10, is essential for epithelial integrity in kidney UB [15]. Furthermore, Rac1 controls the actin cytoskeleton to prevent misshapen cells from entering the cell cycle during renal tubule repair [16].

UB tips reciprocally interact with the surrounding mesenchyme to govern organ‐specific branch patterns [1, 2]. Several growth factors expressed by the metanephric mesenchyme activate receptor tyrosine kinase signaling in the UB [17], notably glial cell line‐derived neurotrophic factor (GDNF) [18, 19, 20, 21, 22, 23] and fibroblast growth factor (FGF) family members [24, 25, 26, 27]. GDNF and FGFs activate mitogen‐activated protein kinase/extracellular signal‐regulated kinase (MAPK/ERK), phosphoinositide 3‐kinase/protein kinase B (PI3K/AKT), and phospholipase Cγ (PLCγ) [28]. Of these, MAPK/ERK functions through the RAS–RAF–MEK–ERK cascade to activate transcription factors and protein kinases targeting processes like cellular adhesion through paxillin regulation and actin polymerization via ARP2/3 [29, 30]. MAPK/ERK is essential for normal branching and maintenance of tip identity, likely due to its roles in proliferation, focal adhesions, and adherens junctions [31, 32, 33, 34].

In the developing mammalian kidney, the UB tip‐localized progenitor cells are motile and undergo luminal mitosis [35, 36, 37]. This suggests major cell shape and adhesion changes, which have not been systematically characterized during UB branching morphogenesis. We utilize a machine learning‐based algorithm, ShapeMetrics [38], traction force measurements, and nuclear shape characterization in normal and MAPK/ERK‐deficient UB epithelium, representative of incompetent branching capacity, to demonstrate that remodeling of cell adhesion facilitates branching via changes in size, morphology, and traction stress of the cells within the UB tip niche.

## Results

**Fig. 1:**
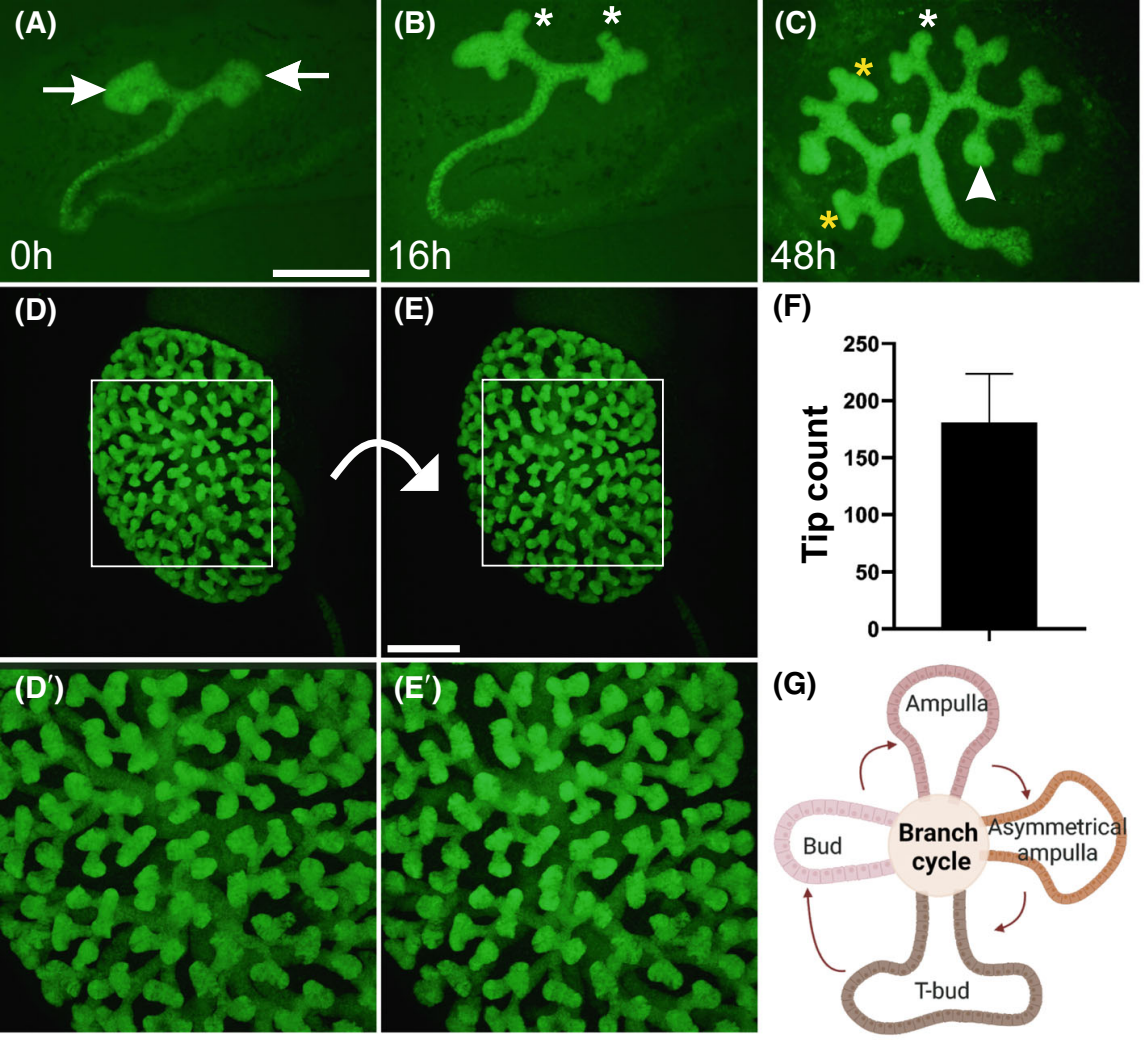
Cellular geometries of ureteric bud epithelium during its branching morphogenesis. E11.5 Hoxb7CreGFP kidney (A) at the start (0 h), (B) 16 h and (C) 48 h of *in vitro* culture. Green fluorescent protein (GFP) visualizes ureteric bud epithelium at its different morphological stages. White asterisk indicates a bud stage, arrowhead points to an ampulla stage, arrow depicts an asymmetric ampulla stage, and yellow asterisks mark T‐bud stage ureteric bud tips. (D, E) Intact E14.5 *HoxB7;Venus* kidney where ureteric bud tree is imaged in 3D and shown from different angles with appr. 35 degrees apart from each other. (D'–E') Zoomed in images of kidneys shown in (D) and (E) visualizing different ureteric bud tip morphologies in a whole‐mount kidney. (F) Quantification of average number of UB tips in kidneys at E14.5 (*n* = 2). (G) Schematic presentation of repetitive ureteric bud branching cycle. Error bars are mean ± standard deviation. Scale bar A–C: 500 μm; D, E: 200 μm.

**Fig. 2:**
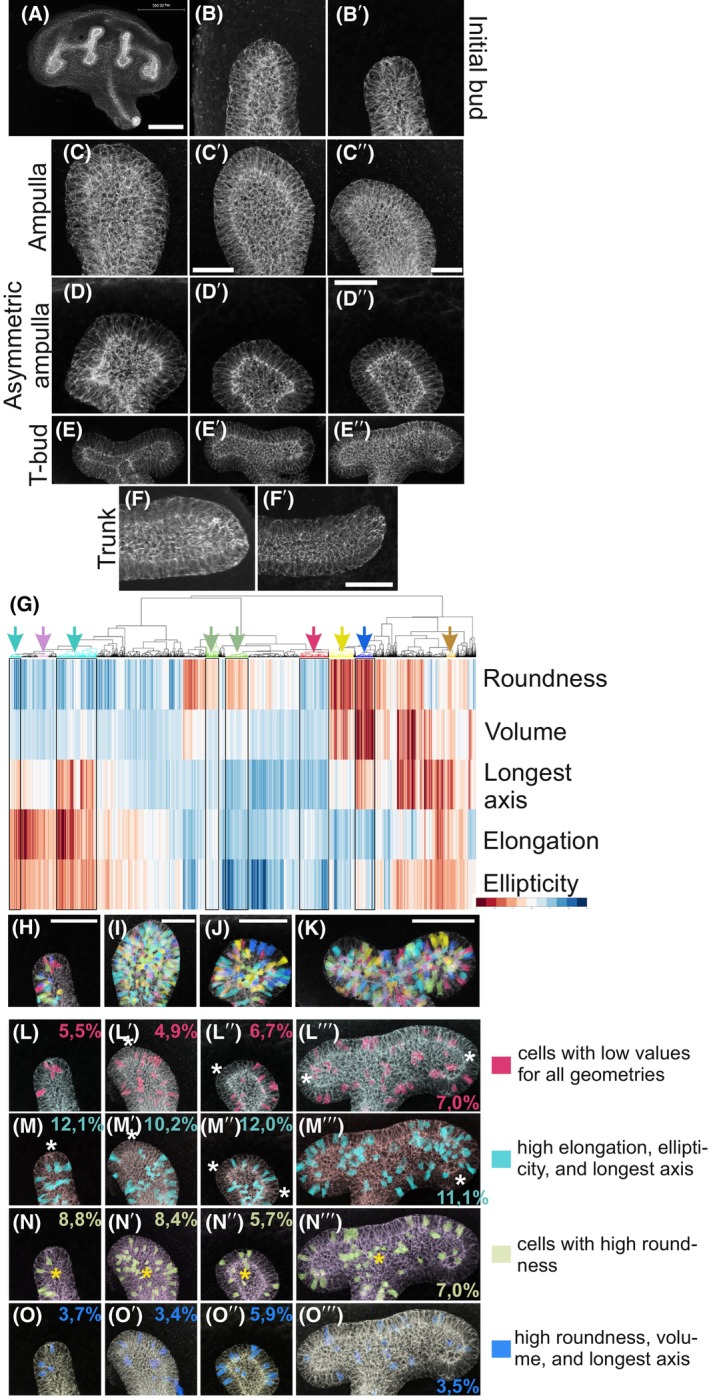
Diverse epithelial cell shapes occupy different tip morphologies of ureteric bud. The ureteric bud epithelium was visualized by E‐CADHERIN staining (white). (A) Representative low magnification image of a kidney at E12.5. The high magnification images of individual (B–B′) initial bud, (C–C″) ampulla stage, (D–D″) asymmetric ampulla, (E–E″) T‐bud stage ureteric bud tips and (F–F′) ureteric trunks. (G) ShapeMetrics supervised machine‐learning‐based algorithm was used to generate a heatmap clustergram of 4437 segmented ureteric bud cells in all branch cycle stages (G) of three different E12.5 mouse kidneys. Each column represents a single cell, the color indicates the measured value of a given geometrical parameter (blue: low; red: high), which are listed on the right (roundness, volume, longest axis, elongation, ellipticity). Boxed areas are indicated by the arrows on top of the image and highlight selected cell clusters, which are mapped back with corresponding colors to original biological samples of (H) initial bud, (I) ampulla, (J) asymmetric ampulla, and (K) T‐bud stage ureteric bud epithelium. Cells in the clusters indicated by black rectangles in (G) are mapped back to original samples at different branch cycle stages. Red cluster: low values for all geometrical parameters mapped back in the (L) bud, (L′) ampulla, (L″) asymmetric ampulla, and (L″′) T‐bud stage ureteric bud epithelium. Turquoise cluster: high elongation, ellipticity, and longest axis, mapped back to (M) bud, (M′) ampulla, (M″) asymmetric ampulla, and (M″′) T‐bud stage ureteric bud epithelium. White asterisks indicate the most cortical UB tip regions. Green cluster: mapping back cells with high roundness to (N) bud, (N′) ampulla, (N″) asymmetric ampulla, and (N″′) T‐bud stage ureteric bud epithelium. Yellow asterisks mark the epithelial lumen. Blue cluster: mapping back epithelial cells with high roundness, volume, and longest axis to (O) bud, (O′) ampulla, (O″) asymmetric ampulla, and (O″′) T‐bud stage ureteric bud epithelium. Numbers in figs L–O show the average proportion of cells that were quantified in each cluster from all biological samples analyzed. *n* = 272/2 initial buds/2 kidneys, *n* = 1517/3 ampullae/2 kidneys, *n* = 508/3 asymmetric ampullae/2 kidneys, *n* = 1733/3 T‐bud/3 kidneys. Scale bar for (A–F) 200 μm; (H–O) 40 μm.

Ureteric bud (UB) branching in the developing kidney utilizes repeated tip bifurcation followed by tube elongation, forming a complex 3D epithelial tree network. The morphological changes are well described [32, 39]: the initial bud balloons into an ampulla, which grows and converts into an asymmetrical ampulla where bifurcation and new growth directions are determined (Fig. 1A–C). This causes new buds to invade into free space away from the parental duct and neighboring tips. Numerous transitory phases occur, with 3D morphology deviating from basic bud, ampulla, and T‐bud forms in some 200 tips in embryonic day 14.5 (E14.5) kidneys (Fig. 1D–F). For our analyses, UB tips were categorized into four major morphological types: initial bud, ampulla, asymmetrical ampulla, and T‐bud (bifurcated) tip (Figs 1G and 2A–F).

### Highly heterogenous ureteric bud cells undergo specific morphological changes during branching morphogenesis

To assess changes in UB cell sizes and shapes, we analyzed 4437 epithelial cells from three E12.5 mouse embryonic kidneys (Table 1). We quantified the four distinct UB tip categories, namely initial bud (*n* = 2), ampulla (*n* = 3), asymmetric ampulla (*n* = 3), T‐bud (*n* = 3), and additionally cells from the UB trunk (*n* = 2) (Figs 1G and 2A–F), using ShapeMetrics parameters: volume, roundness, elongation, ellipticity, and longest axis. Data from the selected UB tips were concatenated into a joint matrix using hierarchical clustering, revealing high heterogeneity in the UB tips (Fig. 2G). To provide spatial information about cell geometries in their native environment, cells with selected features were mapped back onto the original 3D biological sample images (Fig. 2H–O″′). Based on their qualitative assessment, the epithelial cells originating from the different heatmap clusters display high heterogeneity in general and show low variation in their localization. Cells from the ‘low values for all parameters’ cluster were most abundant at T‐bud stage (7.0%), while cells of high elongation, ellipticity, and longest axis were more abundant throughout the entire branch cycle (12.1%, 10.2%, 12%, and 11.1%, respectively) than cells with any other geometrical features (Fig. 2L–O″′). Round cells were least abundant at the asymmetric ampulla stage (5.7%) (Fig. 2N–N″′), possibly reflecting rare events of luminal mitosis [35, 40]. Cells with high roundness, large volume, and high longest axis were most infrequent at all stages except at the asymmetric ampulla stage (Fig. 2O–O″′). In summary, epithelial cells in different tip morphologies appear very heterogenous in their shapes and localizations.

**Table 1:** Details of ureteric bud types and average numeric values for cells segmented at each given branch cycle stage. Cell #, cell number.

Tip type	UB tips	Cell #	Total cells	Volume	Roundness	Elongation	Ellipticity	Longest axis	Intermediate axis	Minor axis
Initial bud	2	272	4437	79.97	3.27	1.93	0.56	5.76	3.69	2.42
Ampulla	3	1517		100.70	3.02	2.28	0.62	7.50	4.08	2.63
Assymmetric ampulla	3	508		91.05	3.55	2.50	0.63	7.22	3.45	2.49
T‐bud	3	1733		100.55	2.26	2.24	0.60	8.59	4.63	3.25
Trunk	2	407		180.78	2.54	1.83	0.53	10.29	6.63	4.66

### Epithelial cell roundness peaks at the asymmetric ampulla stage, while elongation increases steadily until the start of a new branch cycle

**Fig. 3:**
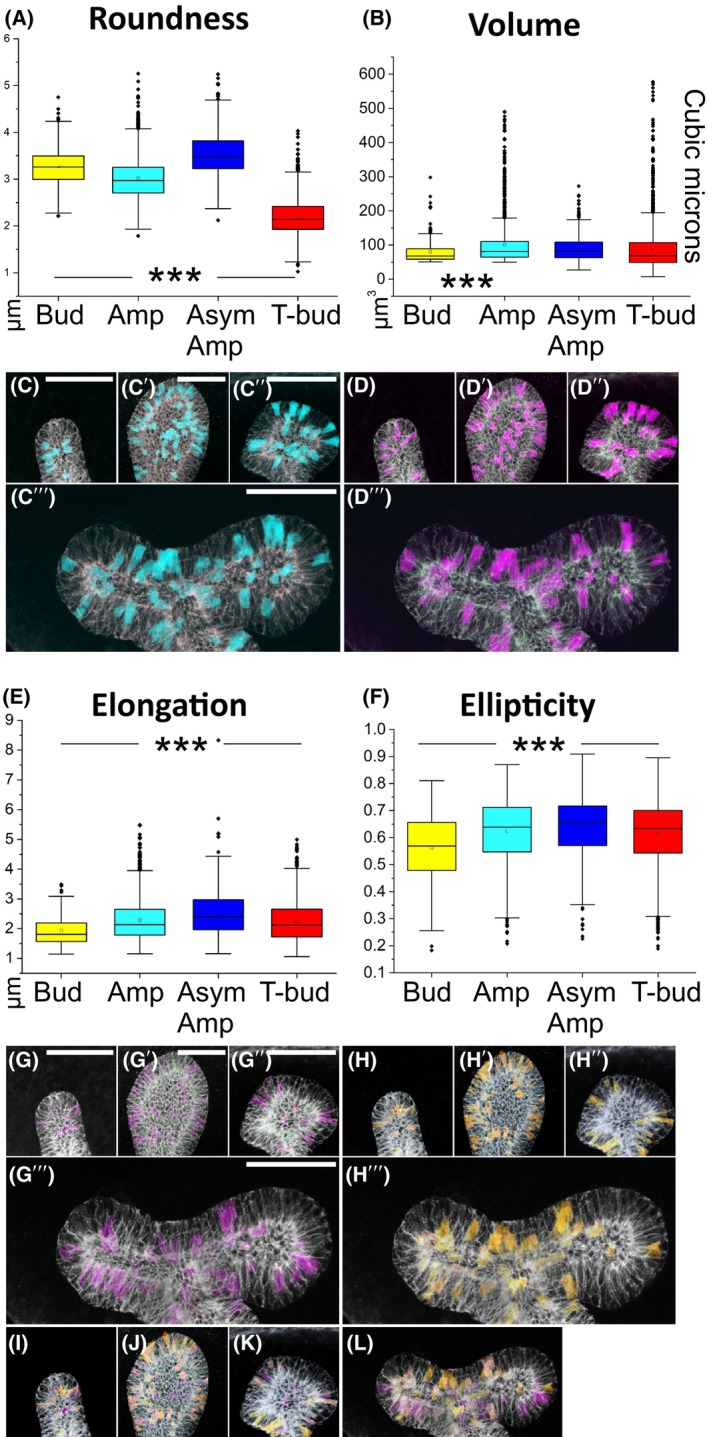
Cells with highest roundness fluctuate between different branch cycle stages while ellipticity increases until asymmetric ampulla. (A) Quantitation of epithelial cell roundness (μm) across different stages of branch cycle demonstrates statistically significant difference between all stages (*P* = 3.96 × 10^−18^, 1.35 × 10^−85^, 0, respectively) and that asymmetric ampulla houses the most round ureteric bud cells. (B) Similar quantitation of volume (μm^3^) shows very little fluctuation between distinct tip morphologies with significant difference only in initial bud‐to‐ampulla stage (*P* = 1.80 × 10^−9^). Top 10% of the most round epithelial cells (turquoise) mapped back to the original biological sample at (C) bud, (C′) ampulla, (C″) asymmetric ampulla and (C″′) T‐bud stage while the top 10% of the high‐volume cells (violet) are shown at (D) bud, (D′) ampulla, (D″) asymmetric ampulla, and (D″′) T‐bud stage. (E) Quantitation of epithelial cell elongation and (F) ellipticity across different stages of ureteric bud branch cycle demonstrates significant differences between all stages (*P* < 0.005, see Table 2 for details). Top 10% of the most (G–G″′) elongated (purple) and (H–H″′) elliptical epithelial cells (green) mapped back to the original kidney sample at bud, ampulla, asymmetric ampulla, and T‐bud stages, respectively. Rainbow overlay of top 10% most elongated (red) and elliptical (yellow) cells at (I) bud, (J) ampulla, (K) asymmetric ampulla and (L) T‐bud stage of ureteric bud demonstrates that many of the cells with highest values for elongation and ellipticity are the same cells. Amp, ampulla stage; As amp, asymmetric ampulla; Bud, initial bud; T‐bud, T‐bud stage. Cells analyzed: *n* = 272/2 initial buds/2 kidneys, *n* = 1517/3 ampullae/2 kidneys, *n* = 508/3 asymmetric ampullae/2 kidneys, *n* = 1733/3 T‐bud/3 kidneys. Statistical significance tested by ANOVA and represented by an asterisk, where *** is *P* < 0.001. Error bars are mean ± standard deviation. Scale bar: C–D, G–L 40 μm.

Quantification of cell features identified roundness as the parameter with the most fluctuation across the branch cycle (Table 1 and Table S1). Roundness was high at the bud stage, diminished in the ampulla, and then peaked in the asymmetric ampulla, showing significant change at each transition (Fig. 3A, Table 2). The most significant increase in roundness occurred between ampulla‐to‐asymmetrical ampulla stages (*P* = 1.34 × 10^−85^), while the biggest drop was between the asymmetrical ampulla and bifurcated tip (Fig. 3A, Table 2), suggesting major morphological changes during this transition. Changes in roundness do not correlate with cell volumes, which remained virtually unchanged from ampulla to T‐bud stage (Fig. 3B), demonstrating a major cell size increase between the initial bud and ampulla stage and suggesting that roundness changes in other tip morphologies are not simply due to cell enlargement. Cell volume increases significantly from bud to ampulla (*P* = 1.79 × 10^−9^) but is similar at all other UB tip stages (Tables 1 and 2). Mapping back the top 10% roundest and highest volume cells showed that they had a similar overall localization pattern in the UB epithelium across the different tip morphologies (Fig. 3C,D).

**Table 2:** *P* values of pairwise statistical testing of difference in given geometrical parameter between adjacent branch stages during cycle progression.

Geometry	Bud‐ampulla	Ampulla‐Assymmetric ampulla	Assymmetric ampulla – T‐bud	T‐bud – bud
Volume	1.80 × 10^−09^	0.3956	0.3956	6.22 × 10^−09^
Roundness	3.95 × 10^−18^	1.34 × 10^−85^	0	1.74 × 10^−265^
Elongation	1.03 × 10^−16^	3.94 × 10^−09^	4.15 × 10^−12^	2.02 × 10^−14^
Ellipticity	9.62 × 10^−14^	0.0682	6.49 × 10^−08^	2.65 × 10^−06^
Longest axis	1.12 × 10^−39^	0.1621	7.02 × 10^−35^	2.98 × 10^−103^
Intermediate axis	2.58 × 10^−11^	1.70 × 10^−43^	2.47 × 10^−139^	4.99 × 10^−55^
Minor axis	1.76 × 10^−06^	4.68 × 10^−06^	7.68 × 10^−125^	1.69 × 10^−87^

**Fig. 4:**
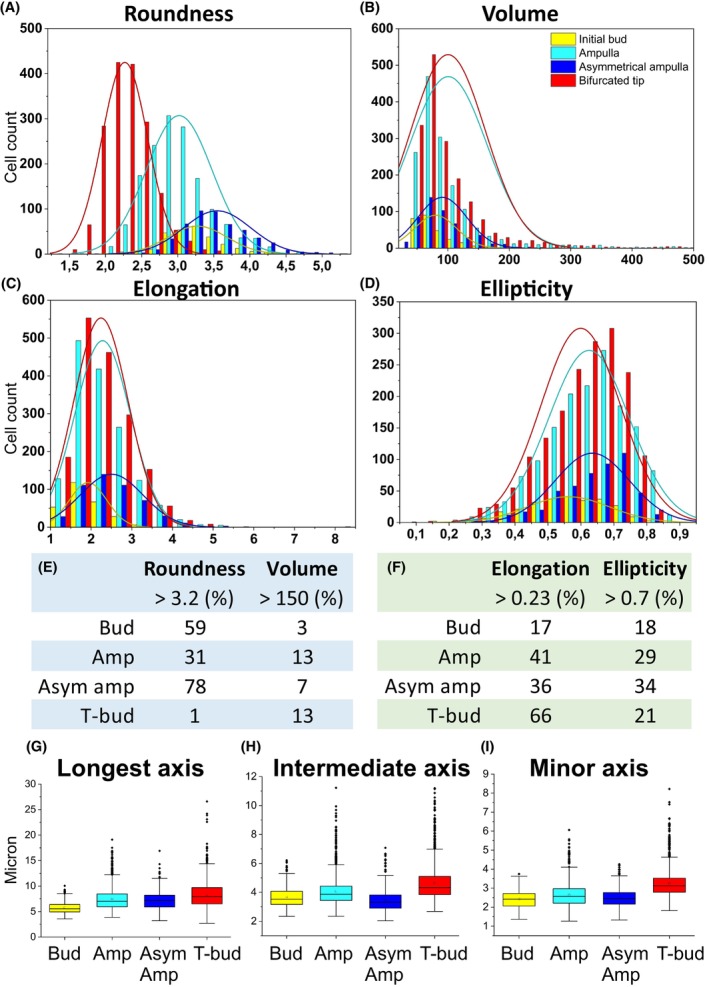
Overlay of distribution plots within the whole volume range (cell count on *Y* axis; (μm) per (μm^3^) on *X* axis) cells. Changes in cellular (A) roundness (μm), (B) volume (μm^3^), (C) elongation, and (D) ellipticity of ureteric bud epithelial cells across the different branching cycle stages are shown. Changes in distributions between tip morphologies were significantly different only for volume (*P* = 7.33 × 10^−5^) and elongation (*P* = 3.38 × 10^−8^) at bud‐to‐ampulla, and for volume (*P* = 0.0027) and roundness (*P* = 2.2 × 10^−16^) at asymmetric ampulla to T‐bud comparisons (Levene's test). (E) Summary of representation (percentage) of ureteric bud epithelial cells with higher than 3.2 roundness and larger than 150 μm^2^ volume during the progression of ureteric bud branch cycle. (F) Summary of representation (percentage) of epithelial cells with elongation higher than 0.23 and ellipticity greater than 0.7 during the progression of ureteric bud branch cycle. Quantification of (G) longest, (H) intermediate, and (I) minor axis in ureteric bud epithelial cells across different stages of ureteric bud branch cycle. Amp, ampulla stage; As amp, asymmetric ampulla; Bud, initial bud; T‐bud, T‐bud stage. *n* = 272/2 initial buds/2 kidneys, *n* = 1517/3 ampullae/2 kidneys, *n* = 508/3 asymmetric ampullae/2 kidneys, *n* = 1733/3 T‐bud/3 kidneys. Error bars are mean ± standard deviation.

Elongation, a parameter calculated as the longest axis divided by the average of the intermediate and minor axis lengths, increased significantly from initial bud until asymmetrical ampulla but decreased again at T‐bud stage (Fig. 3E, Table 2), suggesting that cells elongate the most before the bifurcation. Changes in ellipticity are in line with those of elongation, with the most elliptical cells spatially overlapping with some of the most elongated cells (Fig. 3F–L). Finally, we used distribution plots to further analyze roundness, volume, elongation, and ellipticity. This verified the volume change and additionally identified that the ampulla and T‐bud tip stages appear to occupy a wider volume range (45–485 and 65–500 μm^3^) also including higher volume cells that are not present at asymmetrical ampulla and initial bud stages (Fig. 4A–F). The variance between different branch cycle stages was significantly different only for volume and elongation in initial bud‐to‐ampulla as well as for roundness during asymmetric ampulla‐to‐T‐bud transitions (Levene's test, *P* < 0.005). Interestingly, the axis lengths mimic those detected for elongation and ellipticity with the exception that neither ellipticity nor longest axis is significantly different between epithelial cells at ampulla and asymmetric ampulla stages (Fig. 4G–I, Table 2).

### UMAP clustering of UB tip cells demonstrated the uniqueness of the T‐bud stage

**Fig. 5:**
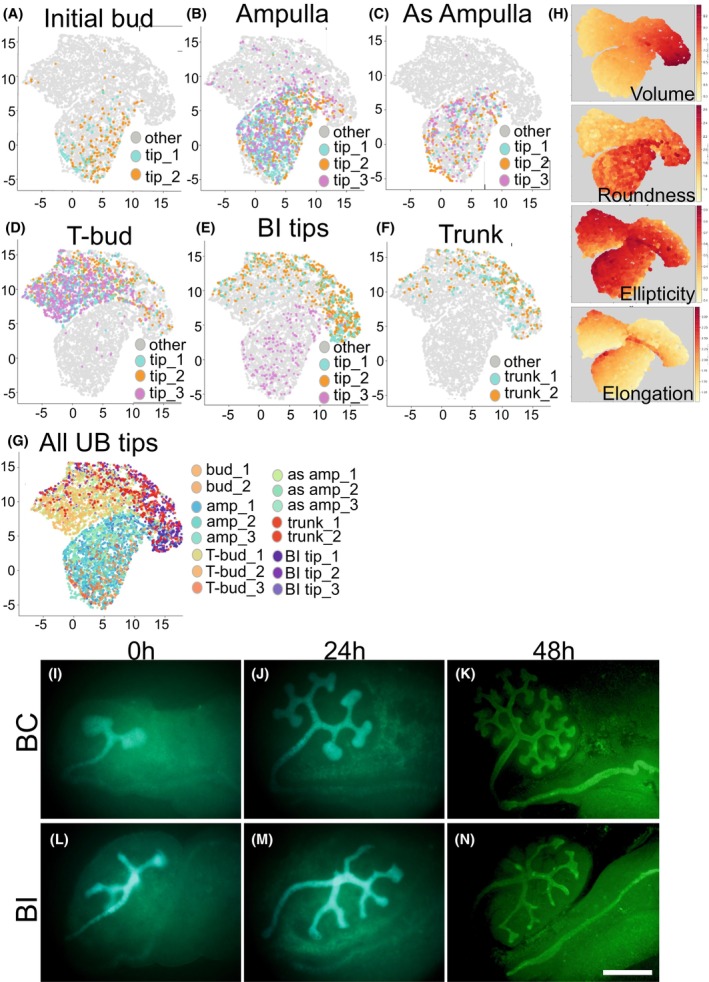
UMAP projection of ureteric bud epithelial cell reveals the individual nature of the T‐bud stage. UMAP of cells segmented at (A) initial bud stage, (B) ampulla stage, (C) asymmetrical ampulla stage, (D) T‐bud stage of branching‐competent (BC), (E) branching‐incompetent (BI) tips, (F) BC trunk and (G) all UB stages together. Cells in each tip are represented with different colors as indicated in each fig. (H) UMAP visualization colored by the levels of each parameter; intense red = high value, light yellow = low value. (I–K) Time‐lapse imaging of *in vitro* cultured BC embryonic kidneys shows the full spectrum of ureteric bud morphological changes as visualized by GFP signal (green) (I, J) and by CALBINDIN immunofluorescent staining (green) at 48 h. (L–N) Time‐lapse imaging of *in vitro* cultured BI (*HoxB7CreGFP;Mek1* ^*fl/fl*^ *;Mek2* ^−/−^) kidneys demonstrates less complex ureteric bud tip structures and impaired growth. Ureteric bud is visualized by Cre‐driven GFP in I‐J and L‐M, and by CALBINDIN staining in K, N leading to slight differences in shades of the green color and better visualization of Wolffian duct. Amp, ampulla stage; As amp, asymmetric ampulla; Bud, initial bud; T‐bud, T‐bud stage. Scale bar: I–N: 500 μm.

Single‐cell Uniform Manifold Approximation and Projection (UMAP) based on geometrical parameters was performed to infer the developmental trajectory of cell shapes in UB tips and trunks (Fig. 5A–H). This demonstrated dynamic changes in elongation throughout UB tip morphogenesis by showing noticeably close clustering of cell geometries at initial bud, ampulla, and asymmetrical ampulla stages. Cells at the initial bud stage localize to UMAP regions showing low elongation. At the ampulla stage, they overlap with both initial bud and asymmetrical ampulla clusters. At the asymmetrical ampulla stage, cell geometries shifted left, representing the most elongated, elliptical, and round cells. The cells in T‐bud tips and UB trunks cluster distinctly from the other UB tip types. They show very low roundness and cluster only with a specific sub‐population of elliptical cells that are not detected in other tip types.

### Cell shape changes in branching‐incompetent UB epithelium

To understand the relationship between cell shapes, geometry, and branching, we studied branching‐incompetent (BI) UB epithelium lacking MAPK/ERK activity (*HoxB7CreGFP;Mek1* ^*fl/fl*^ *;Mek2* ^−/−^), which displays simplified UB arborization [34]. Projecting the cells onto the UMAP plot shows that BI tips are distinct from branching‐competent (BC) tips, which have more complex tip structures (Fig. 5E,F,I–N). UMAP visualization indicates that two out of three BI tips cluster close to UB trunk epithelium and one more closely resembles a BC epithelium at the initial bud stage (Fig. 5E,F). This suggests that epithelial cell morphology in BI tips resembles simpler geometries detected in initial bud stages and trunk epithelium. Interestingly, our previous studies demonstrated premature differentiation of BI tips into collecting duct cell types, supporting morphological similarity to UB trunk cells [33].

**Fig. 6:**
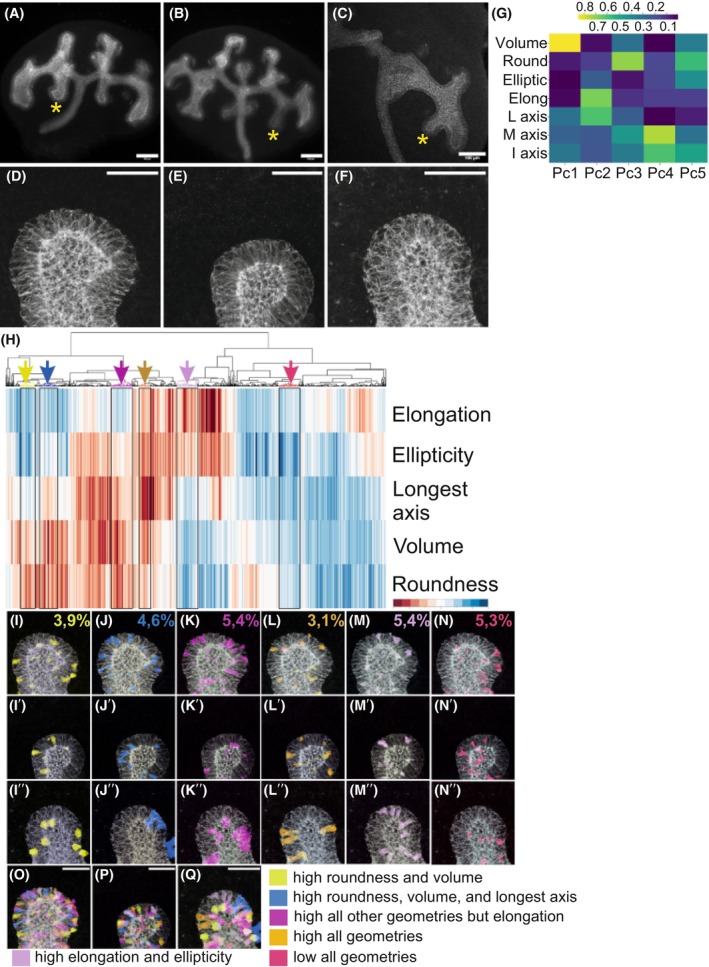
Overall morphology of tips and cell shapes in branching‐incompetent ureteric bud epithelium. (A–C) Three distinct branching‐incompetent E12.5 whole kidneys derived from genetic loss‐of‐function of ureteric bud specific MAPK/ERK activity (*HoxB7CreGFP;Mek1fl/fl;Mek2* ^−/−^) were subjected to E‐CADHERIN staining (white) and used for ShapeMetrics segmentation analyses. (D) High magnification image of branching‐incompetent ureteric bud tip denoted by asterisk in A, (E) in B, and (F) in C. (G) Principal component (Pc) analysis of defining features shown in UMAP in the main Fig. 3. (H) ShapeMetrics algorithm generated heatmap cluster gram of 958 segmented ureteric bud cells in three different branching‐incompetent E12.5 mouse kidneys. Cells are clustered according to their distinct features, and each vertical column represents a single cell. The color of the column indicates the measured value of given geometrical parameter (blue: low; red: high), which are listed in right (elongation, ellipticity, longest axis, volume, roundness). Boxed areas with colored arrows (yellow, blue, violet, brown, lilac, red) highlight the selected cell clusters that are mapped back to original biological samples with corresponding colors in I–N″. (I–I″) Yellow cluster contains cells that are round and large in volume, (J–J″) blue cluster is otherwise similar but has higher longest axis, (K–K″) purple cluster cells have rather high values for all other parameters but elongation, (L–L″) brown cluster cells show high values for all parameters (M–M″) lilac cluster contains cells that are elongated and elliptical, and (N–N″) red cluster cells that show low values for all parameters. Numbers in figs I–N show the average proportion of cells that were quantified in each cluster from all biological samples analyzed. (O–Q) Images showing overlay of all the selected cell clusters in the original kidney samples. Elliptic, elliptical; Elong, elongated; I axis, intermediate axis; L axis, longest axis; M axis, minor axis; round, roundness. Scale bar: (A–F) 500 μm; (I–Q') 40 μm.

The heatmap of three BI tips which exhibit morphologically immature ampullae demonstrated divergent cell shapes (Fig. 6A–H). Mapping cells of different geometries back to original sample images revealed that BI tip cells with high volume and roundness sparsely distributed and primarily located on the basal side of the epithelium, showing variation in their intraepithelial positions (Fig. 6I–N). In general, the BI epithelial cell characteristics showed a similar level of cell morphology and localization variation as seen for BC cells (Fig. 6K–N). Overlaying all subclustered epithelial cell groups demonstrated greater segregation of geometrical parameters in BI than in BC UB epithelium without localization preference for any particular cell morphology (Fig. 6O–Q, see Fig. 2H–K for comparison).

### Branching‐incompetent ureteric bud epithelial cells are high in volume and fail to transform into elliptical shapes

The BI UB epithelium demonstrates less complex tip morphologies, prompting a comparison of general cell geometries with BC epithelia. Global quantification identified cell volume as a major difference between BC and BI UB tip cells (Tables 1, 3 and 4, and Table S1). Remarkably, the cellular volumes in BI tips were significantly larger and showed more than 2.5 times higher volumes in the BC tips (Tables 1, 3 and 4). The average roundness of BI cells (3.07 μm) was also significantly higher than in BC epithelium at the initial bud stage (2.92 μm) but resembled that of BC cells at the ampulla stage (3.02 μm, Tables 1, 3 and 4) while being again significantly lower than BC in cells at the asymmetric ampulla stage (3.55 μm, Tables 3 and 4) when UB tip cells have the highest proliferation index [18].

**Table 3:** Details of branching‐incompetent (BI) ureteric buds and the average numeric values for their cell geometry parameters. As. ampulla, asymmetric ampulla; Cell #, cell number; Ellip, ellipticity; Elong, elongation; I, intermediate; L, longest; M, minor; Round, roundness; Tot cells, total number of cells; Vol, volume.

Tip	Cell #	Tot cells	Vol	Vol	Round	Round	Elong	Elong	Ellip	Ellip	L axis	L axis	I axis	I axis	M axis	M axis
1	501	958	222.24	201.25	2.97	3.07	1.80	1.93	0.50	0.54	9.94	9.49	6.44	5.91	4.69	4.18
2	257		233.62		2.88		1.90		0.53		10.58		6.44		4.71	
3	200		107.09		3.56		2.31		0.65		6.95		3.90		2.22	

**Table 4:** *P* values of pairwise statistical testing of difference in cell geometries between branching‐incompetent (BI) epithelia and initial bud, ampulla, asymmetric ampulla, T‐bud, and trunk, respectively. as. ampulla, asymmetric ampulla.

Geometry	Bud‐BI	Ampulla‐BI	Assymmetric ampulla‐BI	T‐bud‐BI	Trunk‐BI
Volume	1.79 × 10^−79^	2.25 × 10^−102^	1.80 × 10^−70^	4.56 × 10^−111^	0.3956
Roundness	6.98 × 10^−16^	0.6531	9.98 × 10^−73^	0	1.87 × 10^−78^
Elongation	0.5964	4.98 × 10^−46^	6.54 × 10^−60^	4.70 × 10^−41^	0.0683
Ellipticity	0.0682	7.03 × 10^−54^	7.25 × 10^−42^	4.65 × 10^−30^	0.0584
Longest axis	3.13 × 10^−122^	2.25 × 10^−76^	2.14 × 10^−52^	1.19 × 10^−07^	3.18 × 10^−11^
Intermediate axis	1.21 × 10^−152^	5.83 × 10^−236^	5.51 × 10^−294^	3.27 × 10^−91^	2.57 × 10^−20^
Minor axis	3.75 × 10^−168^	1.05 × 10^−303^	1.89 × 10^−233^	1.43 × 10^−56^	8.29 × 10^−23^

**Fig. 7:**
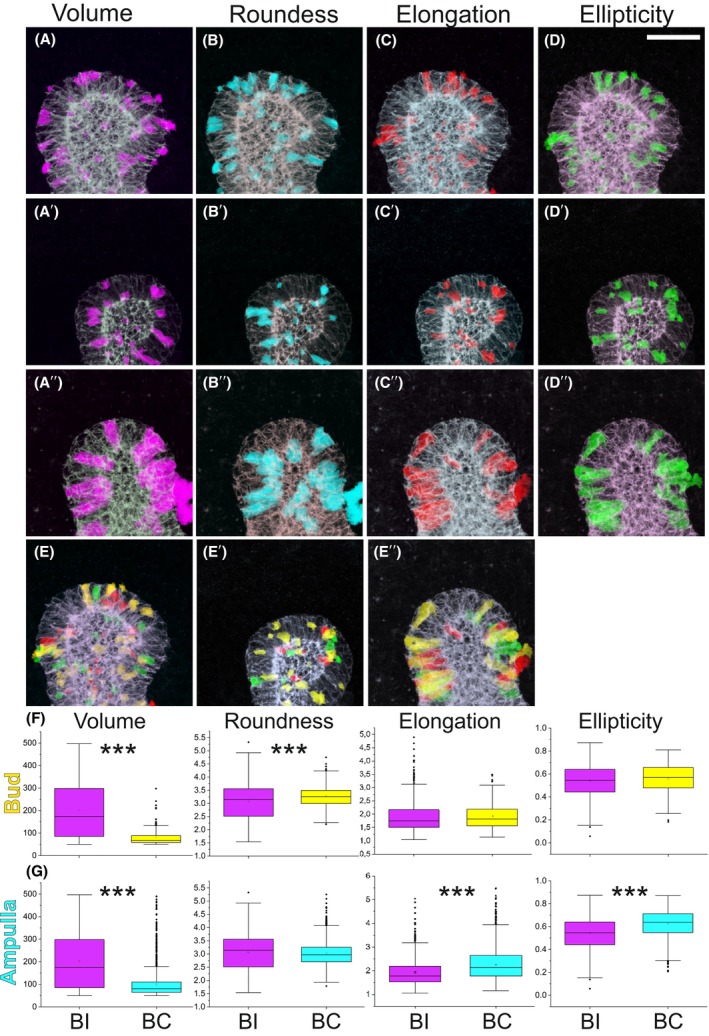
Branching‐incompetent ureteric bud epithelial cells are high in volume. (A–A″) Top 10% of the high‐volume epithelial cells (purple, μ^3^) mapped back to three distinct branching‐incompetent (BI) ureteric bud. (B–B″) Top 10% of the most round epithelial cells (turquoise) mapped back to the three biological replicates of BI epithelium. (C–C″) Top 10% of the most elongated epithelial cells (red) mapped back to the three biological replicates of BI epithelium. (D–D″) Top 10% of the most elliptical epithelial cells (green) mapped back to the three biological replicates of BI epithelium. (E–E″) Overlay of the top 10% most elongated and elliptical cells localization in the three biological replicates of BI epithelium. (F) Comparison of epithelial cell geometries in branching‐competent (BC, yellow) at initial bud stage to‐incompetent (BI, violet) ureteric bud. Whisker plots showing volumes(μm^3^), roundness(μm), elongation and ellipticity in epithelial cells of BC (yellow bars) and BI (violet) ureteric bud demonstrate statistically significant difference in epithelial cell volume (*P* = 1.80 × 10^−79^) and roundness (*P* = 6.98 × 10^−16^) between the two types of epithelia. (G) Comparison of epithelial cell geometries in (BC, turquoise) at ampulla stage to (BI, violet) ureteric bud. Similarly as seen at earliest stage of branch cycle, epithelial cell volume remains significantly higher in BI than BC ureteric bud tips (*P* = 2.25 × 10^−102^). Additionally, while epithelial cell roundness is higher in BI ureteric buds (violet bars) than those in BC (blue) buds, it is not statistically significant (*P* = 0.65). However, the elongation (*P* = 4.98 × 10^−46^) and ellipticity (*P* = 7.03 × 10^−54^) are significantly smaller in BI tips. BC, branching‐competent; BI, branching‐incompetent. Cells analyzed: *n* = 272/2 initial buds/2 kidneys, *n* = 1517/3 ampulla/2 kidneys, *n* = 508/3 asymmetric ampulla/2 kidneys, *n* = 1733/3 T‐bud/3 kidneys. Statistical significance tested by ANOVA and represented by an asterisk, where *** is *P* < 0.001. The data distribution is presented by box plot with whiskers. Scale bar: 40 μm.

Examining the 10% highest volume and roundest epithelial cells indicates that different geometric conformations exist in high‐volume cells (Fig. 7A,B). Some of the top 10% elliptical and elongated cells have large volumes as seen by their overlap, and thus likely compose a portion of the highest volume cells in BI epithelium (Fig. 7C–E). Comparison across the entire branch cycle highlights statistically significant differences in cell volumes between BI and BC epithelium (Table 4), as well as dynamic ascending variations in roundness, ellipticity, and elongation (Fig. 7F,G).

**Fig. 8:**
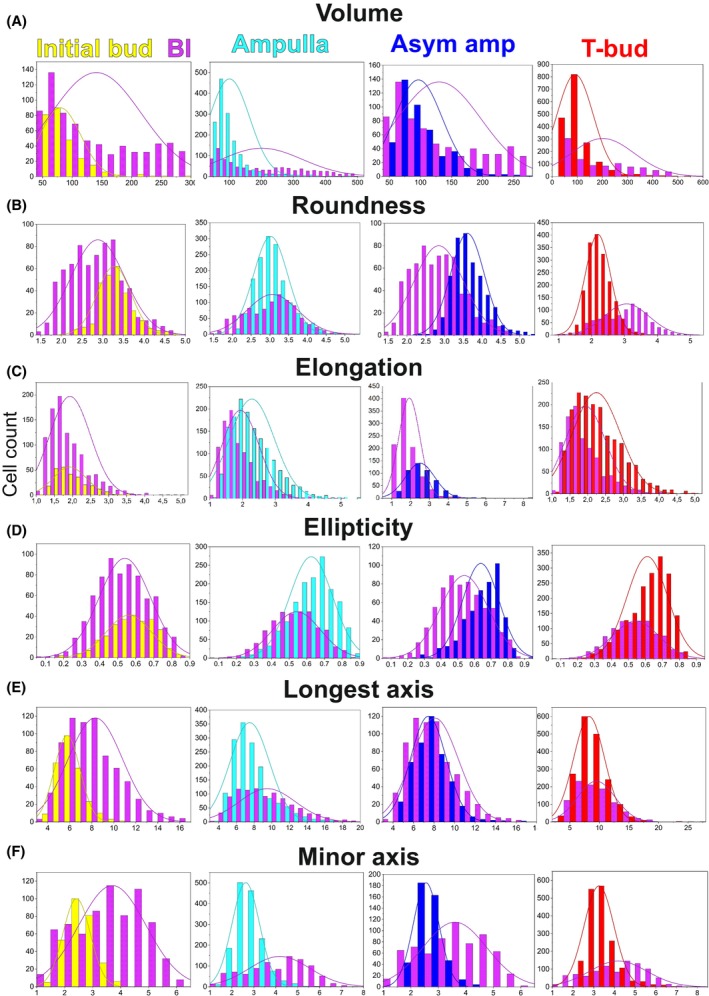
Branching‐incompetent epithelial cells are in general wide and less elliptical than epithelia in branching‐competent kidneys. Distribution plots of cells demonstrate the range where epithelial cell (A) volume (μm^3^), (B) roundness (μm), (C) elongation, (D) ellipticity, (E) longest axis (μm), and (F) minor axis (μm) land in branching‐incompetent (BI, violet) epithelial in comparison to branching‐competent (BC) epithelia at initial bud (yellow), ampulla (blue), asymmetric ampulla (dark blue) and T‐bud (red) stage. The BI epithelial cells show in general larger cell volumes, smaller roundness, and higher longest and minor axis lengths (Levene's test *P* = 2.2 × 10^−16^) than cells at initial bud while the distribution of other geometrical features overlaps quite well. For distribution plots the cells within the shared volume range were used: of 50–298 μm at initial bud (yellow), 50–490 μm^3^ at ampulla, 50–272 μm^3^ at asymmetric ampulla and ~ 50–500 μm^3^ at T‐bud stage. Cells analyzed: *n* = 272/2 initial buds/2 kidneys, *n* = 1517/3 ampulla/2 kidneys, *n* = 508/3 asymmetric ampulla/2 kidneys, *n* = 1733/3 T‐bud/3 kidneys. The range distribution of each parameter in BI cells differs significant from that detected in BC cells (Levene's test *P* = 3.81 × 10^−5^ to 2.2 × 10^−16^). Asym amp, asymmetric ampulla; BI, branching incompetent.

To avoid bias from volume range differences for initial bud and asymmetrical ampulla stages (Tables 1 and 3), comparisons to BI tips were done proportionally and within the common cell volume range. Overlay of and comparison to the ranges in two different types of epithelia show that most cells in the control epithelia are small in volume and have short longest axes. The range differences in cellular geometries between BI and BC epithelia indeed differ significantly in all other tip morphologies (Levene's test, *P* < 0.005) but elongation (*P* = 0.056) and ellipticity (*P* = 0.0062) at initial bud stage (Fig. 8). This showed that the volume range of cells in BI tips is remarkably wider than in BC tips at any stage (Fig. 8A). The distribution of cell roundness in BI tips almost completely overlapped with the roundness distribution in BC tips at the ampulla stage, being only slightly narrower when compared at the initial bud stage (Fig. 8B). In contrast, the asymmetrical ampulla BC epithelial cells generally displayed higher roundness than BI tip cells, but the situation is reversed when BI tip cells are compared to BC T‐bud cells (Fig. 8B).

Ellipticity and elongation values for cells in BI tips resembled the values for BC epithelial cells at the initial bud stage but show a steady decline in later stages (Fig. 8C,D). This suggests possible defects in compressing cuboidal cells into a wedge‐shaped elliptical/elongated forms through the process involving apical constriction [41, 42]. We measured the longest, intermediate, and minor axes and found that median values were statistically significantly higher (*P* < 0.005) in BI than in BC cells at any branch cycle stage (Tables 1, 3 and 4). Stage‐by‐stage comparison of the longest axis shows similar distributions in the BC and BI epithelial cells at asymmetric ampulla and T‐bud stages (Fig. 8E). The BI epithelial cells, however, had significantly lengthier longest axes at the initial bud and ampulla stages, while the minor axes of BI epithelial cells were consistently longer than in BC cells at all branch stages (Fig. 8F).

These results demonstrate that cells in the same volume range in BI epithelia are wider than BC cells at initial bud and ampulla stages. This is verified by the significant differences in their maximal axis lengths (Levene's test, *P* = 8.46 × 10^−10^ to 2.2 × 10^−16^). Moreover, a wider phenotype, supported by the longer minor axes in BI cells (Levene's test, *P* = 2.2 × 10^−16^) when compared to BC epithelia of any morphology, may leave less room for flexibility, thus suggesting an inability of the epithelium to curve as a mechanism for compromised branching.

### Branching incompetent ureteric bud cells have disrupted lateral adhesions and show compensatory focal adhesion changes

**Fig. 9:**
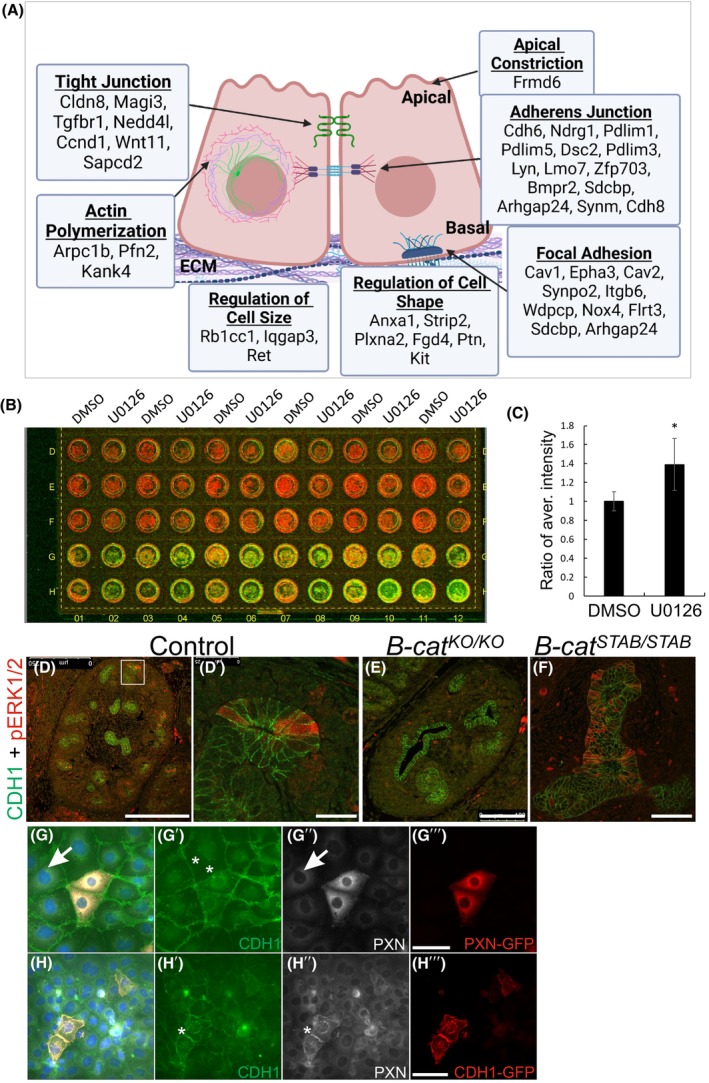
Abnormalities in epithelial cell adhesion are E‐CADHERIN‐dependent in branching‐incompetent ureteric bud epithelium. (A) Graphical illustration of differentially expressed genes (DEGs) in branching‐incompetent ureteric bud epithelium. The cellular localization of DEGs and their association with processes related to adhesion and size/shape regulation is shown by arrows. (B) Representative image of On‐Cell Western results showing fluorescently detected E‐CADHERIN on ureteric bud‐derived epithelial cell surfaces. Every other column is control (DMSO) or MEK‐inhibited (15 μm U0126). Three upper rows are negative controls without primary antibody while the two bottom rows show immunoreactivity of anti‐mouse E‐CADHERIN on cell surfaces. (C) Quantification of E‐CADHERIN intensity as ratio of intensity average (*Y* axis). E‐CADHERIN intensity is significantly higher on MEK‐inhibited epithelial cell surfaces than on control cells (*n* = 3 per treatment, 3 repeats; *P* < 0.05). Localization of E‐CADHERIN (CDH1, green) and phospho‐ERK1/2 (pERK1/2, red) in E12.5 (D) Hoxb7Cre control, (E) tissue‐specific knockout (KO) of Beta‐catenin (B‐cat), and (F) Beta‐catenin stabilized (stab) kidneys reveals loss of MAPK/ERK activation in the absence of *B‐cat* and premature, increased signal when *B‐cat* is stabilized (*n* = 3 per genotype). (G–G″′) Representative images of PAXILLIN overexpression in ureteric bud epithelial cell line at 48 h timepoint. (G) An overlay of staining with E‐CADHERIN (CHD1, green), PAXILLIN (PXN, white) and green fluorescent protein (red) tagged to overexpressed PAXILLIN. Arrow points to endogenous PAXILLIN expression. (G′) E‐CADHERIN staining shows similar levels of protein in both wild‐type and PAXILLIN overexpressing cells (asterisks). (G″) Immunofluorescent detection and visualization of PAXILLIN only demonstrates both endogenous (arrows) and overexpressed protein. (G″′) Visualization of overexpressed PAXILLIN only by GFP‐antibody staining. (H–H″′) Representative images of E‐CADHERIN overexpression in ureteric bud epithelial cell line at 24 h timepoint. (H) An overlay of staining with E‐CADHERIN (CHD1, green), PAXILLIN (PXN, white) and green fluorescent protein (red) tagged to overexpressed E‐CADHERIN. (H') Immunofluorescent detection and visualization of E‐CADHERIN only shows increased protein in cells with overexpression (asterisk) and normal levels in the cells without overexpression. (H″) Visualization of PAXILLIN only demonstrates intensified protein localization in the epithelial cells overexpressing E‐CADHERIN (asterisk). (H″′) Visualization of overexpressed E‐CADHERIN only by GFP‐antibody staining. Statistical significance analyzed by ttest2 and represented by an asterisk, where * is *P* < 0.05. Error bars are mean ± standard deviation. Scale bar: (D), 250 μm; D', (F), 25 μm; (E), 100 μm, G–H 50 μm.

We have previously reported adhesion abnormalities for the MAPK/ERK‐deficient UB epithelium used here to model disturbed branching [34]. Re‐analysis of MAPK/ERK‐deficient transcriptomic data [33] identified multiple differentially expressed genes (DEG) involved in adherens and tight junctions, focal adhesions, and regulation of cell size and shape (Fig. 9A). As proof of the molecular abnormality of adherens junctions in this model, an overall 40% increase in surface E‐CAD was detected on MAPK/ERK‐inhibited UB‐derived cells (Fig. 9B,C, *P* = 0.002996, *n* = 5). The interplay of MAPK/ERK activity and beta‐catenin, which links adherens junctions to the actin cytoskeleton and is required for UB branching [43, 44], was next determined. Genetic loss of beta‐catenin resulted in reduced MAPK/ERK activity in UB epithelium, while the stabilization of beta‐catenin increased ERK activation (Fig. 9D–F) suggesting a mechanosensitive nature for ERK activation [45] also in the UB epithelium of the developing kidney.

**Fig. 10:**
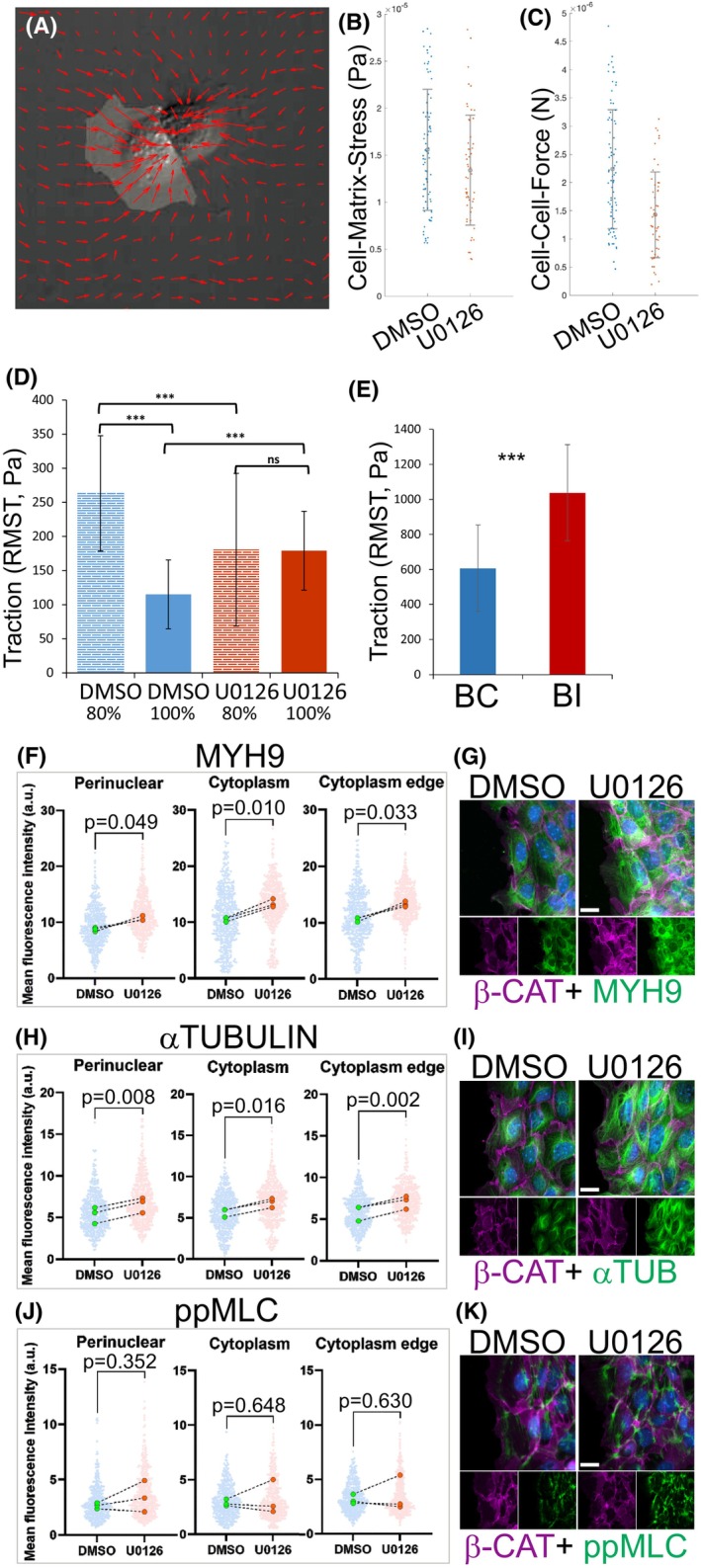
Changes in adhesive forces and Actin dynamics contribute to epithelial cell shape modification and nuclear wrinkling in branching‐incompetent ureteric buds. (A) Demonstrative traction force microscopy image of a cell doublet. Fluorescent bead displacements recorded before and after removal of two adherent cells were processed as described in Materials and methods, yielding traction vectors whose orientation denotes force direction and whose length is proportional to stress magnitude at cell‐substrate and cell–cell interfaces. This image illustrates the appearance of the raw (micrograph of cell doublets) and processed (with red vector map overlay) data. (B) Quantification of stress (Pa) exerted by ureteric bud (UB)‐derived cell doublets treated with U0126/DMSO onto polyacrylic acid matrices (*P* = 0.0511). Measurements were taken from 85 independent doublets across five technical replicates for DMSO control group and from 59 independent doublets across four technical replicates for U0126‐treated group. Error bars are ± standard deviation. Significance was determined by two sample *t*‐test with matlab function ttest2. (C) Quantification of forces (*N*) between UB‐derived cells in doublets treated with U0126 (to mimic branching defect) or DMSO control (*P* = 4.39 × 10^−6^). (D) Average root mean square traction (RMST) forces of UB‐derived cell line at ~ 80% (*P* = 0.000166) and 100% (*P* = 9.59 × 10^−8^) confluence on fibronectin‐coated 25 kPa gels. Measurements were taken from three technical repeats. (E) RMST of primary UB cells isolated from 3 control (BC) and 3 UB‐specific MAPK/ERK‐deficient (BI) embryos at E12 and allowed to delaminate for 48 h on fibronectin‐coated 25 kPa gels before imaging (*n* = 3 per genotype). *P* = 5.69 × 10^−6^. (F) Quantification of mean fluorescent intensities of MYOSIN 9 (MYH9) signal in control (DMSO) and MEK‐inhibited (U0126) primary ureteric bud (pUB) epithelial cells. (G) Representative images of MYH9 (green) and BETA‐CATENIN (b‐CAT, magenta) co‐immunolabeling in control (DMSO) and MEK‐inhibited (U0126) pUB epithelial cells. (H) Quantification of mean fluorescent intensities of alpha‐TUBULIN (aTUB) signal in control (DMSO) and MEK‐inhibited (U0126) pUB epithelial cells. (I) Representative images of aTUB (green) and b‐CAT (magenta) co‐immunolabeling in control (DMSO) and MEK‐inhibited (U0126) pUB epithelial cells. (J) Quantification of mean fluorescent intensities of phosphorylated MYOSIN Light Chain (ppMLC) signal in control (DMSO) and MEK‐inhibited (15 μm U0126) primary ureteric bud (pUB) epithelial cells. (K) Representative images of ppMLC (green) and b‐CAT (magenta) co‐immunolabeling in control (DMSO) and MEK‐inhibited (15 μm U0126) pUB epithelial cells. Measurements were taken from three technical repeats. Statistical significance analyzed by ttest2 and represented by an asterisk, where *** is *P* < 0.001. Error bars are mean ± standard deviation (D, E). Scale bar: G, I, K 20 μm.

The causality and functional consequences of adhesion changes [34] (Fig. 9A) were next assayed. To discern the origin of the adhesion defects, we used overexpression strategies. PAXILLIN overexpression in UB‐derived cells had no effect on E‐CAD, whereas excess E‐CAD provoked similar mislocalization of PAXILLIN on cell membranes (Fig. 9G,H) as documented for BI UB tips *in vivo* [34]. Quantification of BI epithelial cell adhesive forces was carried out to assess functional consequences of altered adhesion molecules. Analysis of UB‐derived cell doublets treated with MAPK/ERK inhibitor U0126 to mimic BI cells revealed a significant decrease in the force between BI epithelial cells (*P* = 4.3940e^−6^), while only a minor change (*P* = 0.0511) in traction stresses exerted by the cells onto the matrices was detected (Fig. 10A–C). Measurement of focal adhesion function demonstrated significantly reduced cell‐matrix stress in MAPK/ERK‐deficient cells at ~ 80% confluence (*P* = 0.000166) (Fig. 10D). However, at 100% confluence and in primary UB cells, increased traction stress towards the matrix was detected (Fig. 10D,E, *P* = 9.59 × 10^−8^, 5.69 × 10^−6^ respectively). Note that the baseline traction for the control group at 80% confluence is nearly three times the baseline for the same group at 100% confluence (*P* = 2.45 × 10^−17^) in control cells, while the traction remained unchanged regardless of confluence in MAPK/ERK‐inhibited UB‐derived cells (*P* = 0.938587). These results show functional deficiency in adherens junctions and focal adhesions, which, along with the actomyosin network, function as biomechanical sensors [46].

Cell adhesions are intricately linked to actomyosin anchoring cadherins at adherens junctions and, together with the microtubule network, contribute to the mechanosensitive regulation of cell shapes [47, 48]. Loss of non‐muscle myosin functions in UB epithelium impairs apical constriction and E‐CAD‐mediated adhesions accompanied by increased ERK activation [15]. To test if actomyosin contractility and/or microtubule networks contribute to cell morphology regulation in UB epithelium, the localization and intensities of MYH9, α‐TUBULIN, and phosphorylated MYOSIN Light Chain (ppMLC) were studied. This showed significantly increased MYH9 (*P* = 0.049, 0.010, and 0.033) and α‐TUBULIN (*P* = 0.008, 0.016, and 0.002) levels in BI cells (Fig. 10F–I), whereas ppMLC (*P* = 0.352, 0.648, and 0.630) levels remained unaffected (Fig. 10J,K). These suggest that MAPK/ERK activity participates in sensing biomechanical cues from the surrounding tissues into UB epithelium, similar to contractile and extensile forces [49, 50].

### Branching‐incompetent ureteric bud epithelial cells display disturbed nuclear morphology and reduced F‐actin rearrangements

**Fig. 11:**
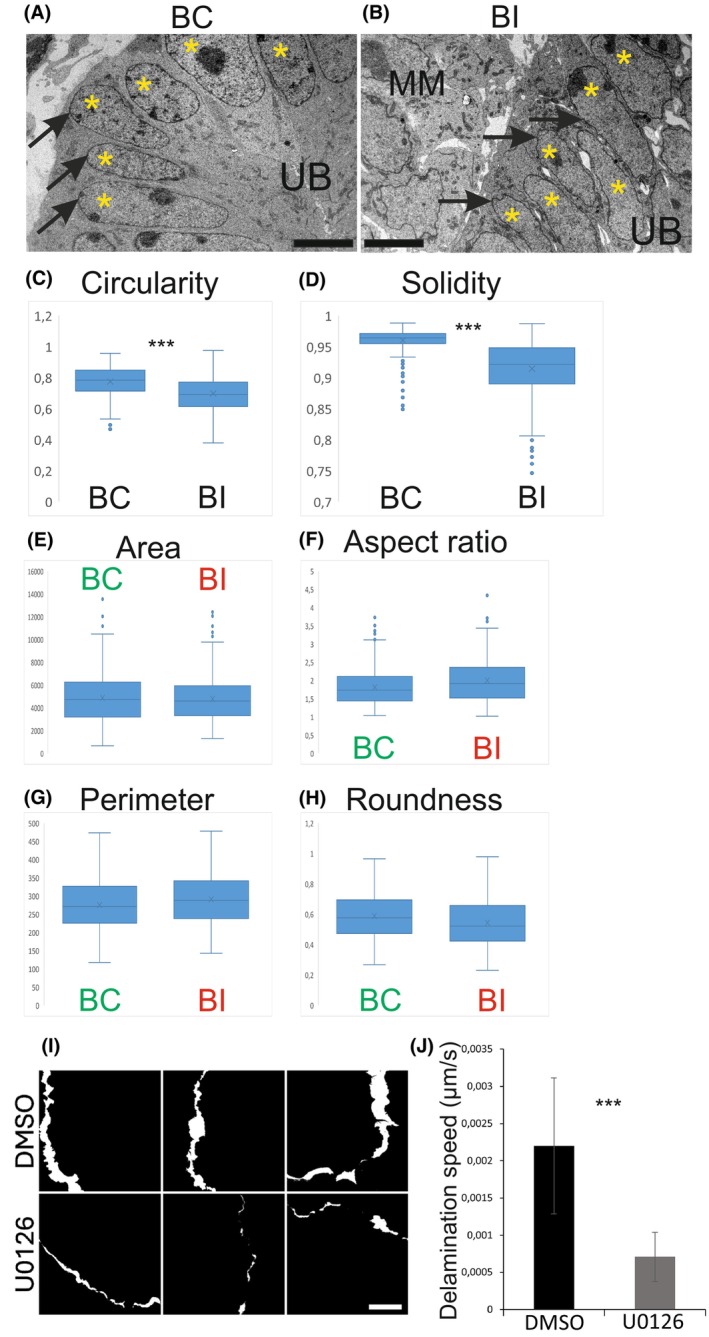
Branching‐compromised ureteric bud tips have wrinkled nuclei. (A) Representative electron microscopy image showing nuclear shapes in branching‐competent (BC) ureteric bud (UB) epithelium of kidneys at E12.5. (B) Representative electron microscopy image showing nuclear shapes in branching‐incompetent (BI) ureteric bud epithelium of kidneys at E12.5. MM shows metanephric mesenchyme, asterisks decipher UB epithelial cell nuclei, arrows point to nuclear envelopes, which are wrinkled and irregularly shaped in BI ureteric buds. (C) Quantification of nuclear circularity, (D) solidity (wrinkling), (E) area, (F) aspect ratio, (G) perimeter, and (H) roundness in the BC and BI ureteric bud epithelial cells show significantly reduced values for circularity and solidity in BI (*P* < 0.001). Measurements were taken from 513 cells in 8 BC and 402 cells in 6 BI kidneys. (I) Quantification of the mean speed of UB delamination (μm·s^−1^) in control (DMSO) and MAPK/ERK‐inhibited (U0126) primary UB epithelial cells measured as the increase in size of delaminating cell masses over 5280 s (88 min) on fibronectin‐coated glass. Images were taken at *t* = 0 and *t* = 5280 s, and binary masks were made from the images and overlaid to show the distance that the cell masses moved during delamination over the 5280 s time period (white parts of the images indicate the areas that the cell masses expanded). Measurements were taken from 17 independent areas from five delaminating primary UBs for DMSO and from five independent areas from two delaminating UBs for U0126. *P* = 2.14 × 10^−5^. Error bars are mean ± standard deviation. (J) Quantification of the mean speed of UB delamination (μm·s^−1^) measured as the increase in size of delaminating cell masses over 5280 s (88 min) on fibronectin‐coated glass. Measurements were taken from 17 independent areas from five delaminating primary UBs for DMSO and from five independent areas from two delaminating UBs for U0126. *P* = 2.14 × 10^−5^. Statistical significance analyzed by ttest2 and represented by an asterisk, where *** is *P* < 0.001. Error bars are mean ± standard deviation. Scale bar: (A, B) 5 μm, (I, J) 50 μm.

Emerging data indicate that changes in cell adhesion are intimately coupled with nuclear morphology and structure [51, 52]. The nucleus acts as a biomechanical sensor of intrinsic and extrinsic mechanical forces. Since BI epithelial cells demonstrate remarkable abnormalities in their adhesion forces, nuclear shapes in wild‐type and BI UB epithelium were analyzed using shape descriptors in fiji (image). This revealed a striking difference in nuclear morphology, as nuclei in BI UBs exhibit a wrinkled nuclear envelope (Fig. 11A,B). Quantification showed no change in size (*P* = 0.613 area, 0.166 perimeter) or roundness (*P* = 0.313) but revealed a significant decrease in the circularity (*P* = 0.0024) and solidity (*P* = 0.00018) of the nuclear membranes in BI epithelium (Fig. 11C–H), indicating increased irregularity of the nuclear boundaries, thus confirming nuclear wrinkling.

Given the role of the actin cytoskeleton in maintaining cellular shape and the effects of ERK on actin polymerization and leading‐edge protrusion during cell motility [30, 53, 54, 55, 56, 57], we studied F‐actin dynamics in UB primary cells bearing the LifeAct‐GFP reporter. Time‐lapse imaging of GFP‐labeled actin at the leading edges of delaminating UBs revealed a defect in actin polymerization and reduced actin dynamics in MAPK/ERK‐deficient cells (Video S1). In addition, the distance of expansion of the leading edges of the delaminating cell masses was calculated over the imaging time to give the average delamination speed (Fig. 11I), detecting that MAPK/ERK‐deficient primary UB epithelial cells delaminated significantly slower (*P* = 2.14E‐05) than the control cells, at approximately one‐fourth of the speed (Fig. 11J).

### Disruption of actomyosin causes tip morphology to resemble branching‐incompetent ureteric bud

**Fig. 12:**
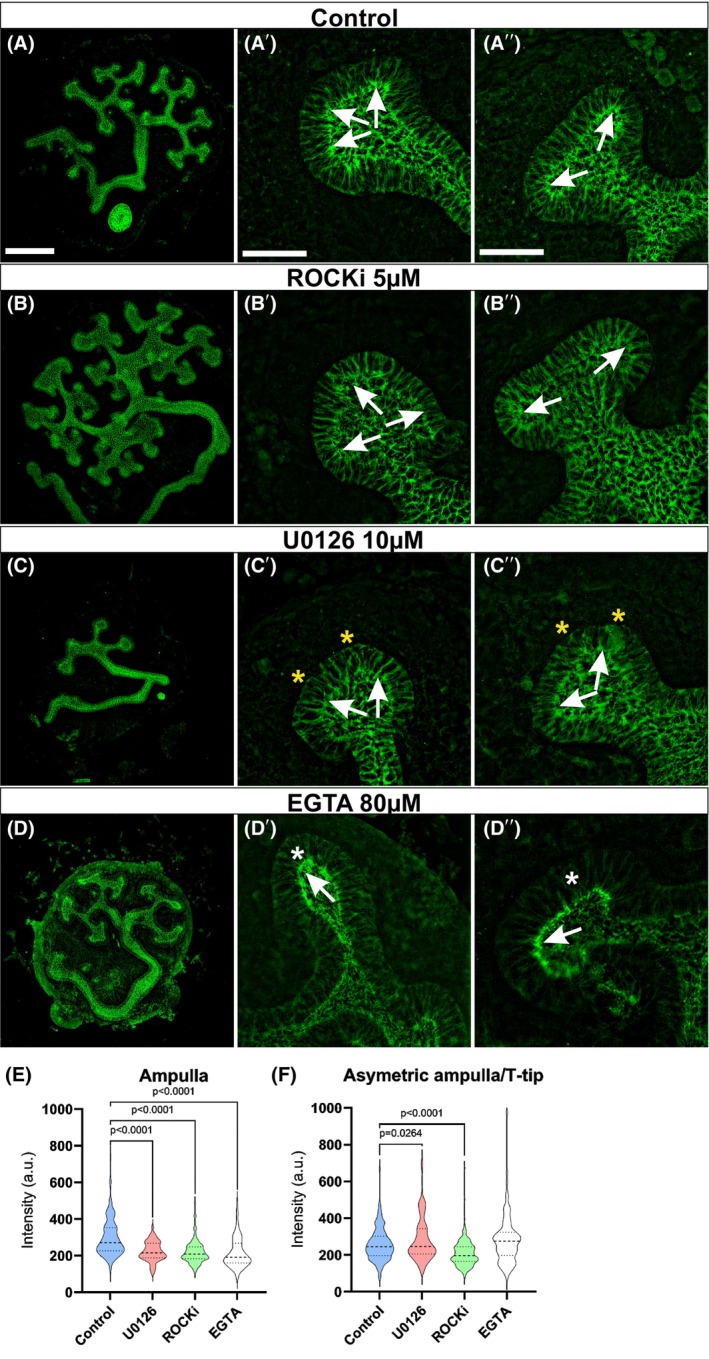
Inhibition of Rho‐kinase and MEK activities disturb apical constriction in branching ureteric bud epithelium. Representative images of E‐CADHERIN (green) stained ureteric bud epithelium in E11.5 wild‐type kidneys, which were cultured for 48 h in the (A) control (DMSO, *n* = 7), (B) 5 μm Rho‐kinase 1 & 2 inhibitor (ROCKi, *n* = 8), (C) 10 μm MEK‐inhibitor (U0126, *n* = 4) and (D) 80 μm egtazic acid (EGTA) (*n* = 4) to disrupt adherens junction dynamics. High magnification of control ureteric buds (A′) at ampulla and (A″) bifurcated bud stage show crowded accumulation of bright E‐CAD foci in the apical surface of epithelium (arrows) indicative of apical constriction, which is distinctive during epithelial bending. A failure to accumulate E‐CAD‐high foci in the apical site of ureteric bud tips regardless of the tip morphology is seen in (B′–B″) ROCKi and in (C′–C″) MEK‐inhibited kidneys, which additionally show E‐CAD accumulation the on basal membranes (asterisks). (D′–D″) E‐CAD staining in EGTA kidneys results in strong apical surface (arrows) staining but weaker signal in intercellular junctions (white asterisk). Quantification of E‐CAD intensities at apical cell membranes of 20 randomly selected cells in (E) ampulla (control *n* = 9, U0126 *n* = 3, ROCKi *n* = 6, EGTA *n* = 4) and (F) T‐bud (control *n* = 9, U0126 *n* = 7, ROCKi *n* = 10, EGTA *n* = 6) stages shows decrease of E‐CAD indicating relaxation in the UB tips with branching defects. Violin plots show median (central dashed line), first (lower dotted line), and third quartiles. Statistical significance analyzed by ttest2. Scale bar A–D: 250 μm, A′–D″: 50 μm. Ureteric bud (UB) branching determines the final size, shape, and nephron number of kidneys. Characterization of UB cell shapes identified dynamic round‐to‐elliptical transition and cellular volume changes as critical epithelial modifications that increase morphological complexity in UB tips during their transitions through ampulla‐to‐asymmetric ampulla conformations. Our findings support the cellular crowding model in transforming individual cells into elliptical and elongated shapes to facilitate diversity in decisions of new branch orientations.

Our characterization of BI epithelium identified significant aberrations in cellular adhesion, actomyosin network, and actin dynamics. Next, we wanted to functionally test how the identified changes affect UB branching. Rho‐associated coiled‐coil‐containing protein serine/threonine kinases ROCK1 and ROCK2 regulate both actin cytoskeleton and actomyosin contractility [58]. Disruption of ROCK1/2 function abolishes normal cytoskeletal and actomyosin functions and, during kidney development, has a pleiotropy of published effects [59, 60, 61, 62]. Application of 5 μm ROCK inhibitor (Y‐27632) to control kidneys resulted in a reported growth‐increasing effect (Fig. 12A,B). The concentration of strong E‐CAD‐positive adherens junctions near the apical surface of epithelial cells detected in control kidneys did not occur in ROCKi UB tip epithelium, where the morphology of the tip became enlarged, indicating failure of apical constriction, as previously shown by actin staining [60].

Levels of MYH9 and αTUB, the principal proteins of the actomyosin network, were increased in BC epithelial cells (Fig. 10F–I). Inhibition of their regulation through ROCK1/2 results in an almost opposite morphological effect compared to those detected in MEK‐inhibited (10 μm U0126) kidneys and in the genetic model of Mek1/2 loss (Fig. 5L–N), which shows thinning of the UB stalk and defective tip enlargement. Like in ROCK‐inhibited kidneys, E‐CAD strong positive foci were not accumulated to apical epithelia in MEK‐inhibited kidneys (Fig. 12C–C″). Disruption of adherens junctions through calcium chelation with 80 μm EGTA treatment, on the other hand, caused UB tips morphologically similar to MEK‐inhibited kidneys with a very strong and thick apical string of E‐CAD (Fig. 12D′–D″). Quantification of apical E‐CAD intensities under different treatments verified our observations (Fig. 12E,F, mean intensity in control cells: 256, MEK‐inhibited cells: 281.3 with *P* = 0.026; Rho‐inhibited cells: 208.4 with *P* < 0.0001; EGTA inhibited: 278.9 with *P* = 0.061) and suggests that UB tips with inhibited Rho‐ and MEK‐kinase activities have less constriction in their epithelial apical surfaces. These organ‐level findings support our cellular findings indicating that defects in cytoskeletal and actomyosin dynamics as well as adhesion force changes contribute to the failure to generate proper ampulla/asymmetric ampulla stages in BI epithelium.

## Discussion

The orchestrated interplay of epithelial branching morphogenesis with surrounding tissues drives coordinated differentiation of many mammalian organs, including the kidney. Cellular processes like bending, folding, lengthening, and narrowing modify cell shapes and volumes to achieve unique branching strategies [1, 2, 63]. UB branching morphogenesis guides kidney development, and while its molecular regulation is well established, cellular mechanisms in new branch point determination are less known [3, 64]. We utilized the AI‐based ShapeMetrics pipeline and Ilastik segmentation of cell borders to analyze UB epithelial cell geometries during different branch cycle stages, including competent (BC) and branching‐incompetent (BI) kidneys with inactivated MAPK/ERK in UB epithelium [34, 38].

Our analysis identified high heterogeneity in epithelial cell geometry throughout all UB branching stages. This heterogeneity is evident from the initial bud stage and remains constant. Geometrical parameters like volume and ellipticity are consistent across UB morphologies, while roundness fluctuates most, and elongation shows a steady increase until the asymmetric ampulla stage. The highest number of cells per tip is at the ampulla stage, suggesting that epithelial crowding precedes new branch site determination. UMAP clustering distinguishes T‐bud stage tips from other stages, indicating their unique characteristics. Hierarchical clustering and cell mapping revealed that elongated and elliptical cells, and cells with low values for all parameters, localize to UB tip regions at the asymmetric ampulla and T‐bud stages, suggesting distinct identities within UB tip domains.

Analysis of cellular properties in BI UB tips identified that cell size (volume) is clearly larger in BI UB epithelium than in any tip type of a normally branching UB. Furthermore, epithelial cells of BI UB tips are on average less elliptical and elongated than those in normally branching kidneys and show statistically significant changes when compared to normal tip morphologies beyond the initial bud stage. Quantitative analysis together with UMAP clustering demonstrated that the shapes of BI cells most resemble those cellular shapes found in initial bud and trunk epithelial cells, an observation that is further validated by the lack of statistical significance in their differences. This is in line with our previous findings showing premature and ectopic differentiation of UB epithelium in BI tips [33]. Control of cell shape changes during branching involves coupling of extrinsic biochemical signals and mechanical forces [65, 66], with changes in intrinsic mechanisms such as cellular adhesion and cytoskeletal rearrangements [47, 67, 68, 69, 70]. Reduced actin dynamics and increased MYH9 and αTUB levels in BI epithelium suggest defects in cytoskeletal processes that are required not only for branching in general [48, 63, 70] but also for individual cell geometry. Microtubules and the actomyosin network resist compressive forces and maintain shapes, especially in large cells [71]. Increased myosin and αTUB indicate that BI epithelial cells are rigid and thus more resistant to dynamic adaptation of cell size based on biochemical and mechanical stimuli.

Distinct mammalian cell morphologies reflect their specialization and often the control of cell shape is coupled to cell fate decisions [72]. Interestingly, small cell size has been linked to stemness in skin and the hematopoietic system, where bigger sized cells show reduced proliferation capacity and increased terminal differentiation [73, 74, 75]. Our previous transcriptomic data demonstrated that BI epithelium loses UB tip identity and prematurely differentiates into collecting duct cells [33], which, together with segmentation data, suggest that cell size could also reflect its differentiation status in the developing kidney.

We previously reported that BI epithelia have abnormalities in E‐CAD‐mediated adherens junctions and in VINCULIN/PAXILLIN at focal adhesions [33, 34]. Here we show that E‐CAD is significantly increased in cell membranes upon MAPK/ERK inhibition. This may reflect differences in E‐CAD protein abundance and/or in accessibility of the E‐CAD to antibodies, suggesting functional adhesion defects. Indeed, we detected significant changes in both cell–cell adhesion force and cell‐matrix traction stress. Since adherens junction‐cytoskeletal linkage couples actomyosin and focal adhesions to cell–cell communication and functions as an essential mechanosensitive sensor [76] this result suggests that BI epithelial cells may have altered responses to biomechanical stimuli. This is supported by our findings demonstrating highly wrinkled nuclear shapes in the BI UB tips, as it has been shown that altered biomechanical forces from the ECM onto cells affect nuclei [52]. Thus, nuclei of the branching epithelium in the developing kidney appear as dynamic organelles capable of mechanosensing and rapid remodeling in response to changes in cellular adhesive properties.

Kidney explants and UB cultures suggest that polarized actin localization and myosin‐based apical constriction influence UB branching morphogenesis [59, 60, 77]. RAS‐MAPK signals activate the actomyosin cytoskeleton for apical constriction, crucial for developing lungs, salivary glands, and mature renal collecting ducts [41, 42, 78, 79]. This activation does not occur in BI epithelial cells, which lack MAPK/ERK activation due to *Mek1/2* deletion [34] likely explaining why BI epithelial cells are large in volume and show significant changes in axis lengths, eventually converting into less elongation and ellipticity than BC cells in the tip morphologies beyond the initial bud stage. MAPK/ERK activity is required for cell cycle progression, and our previous studies have shown that BI epithelial cells fail to progress through the G1/S transition at a similar pace as control cells [34]. This may partially explain why BI cells remain high in volume since cells increase their organelles and volume throughout the G1 phase. Overall, our findings propose a proliferation‐ and stretching‐based model for new branch formation in developing kidneys, as highlighted in the schema. Normally, cells increase in number due to high proliferation [18], causing crowding at the ampulla stage. Morphogenesis from ampulla to asymmetric ampulla involves oriented cell stretching to acquire more elongated and elliptical epithelial cell shapes that allow their transformation into columnar shapes. Cell shape changes facilitate tissue bending and curvature formation, which, in the developing lung, appear to be MAPK/ERK‐dependent [80, 81]. We show here that such changes in cell geometry do not occur in branching‐incompetent UB tips, which simultaneously have adhesion and cytoskeletal abnormalities that make individual epithelial cells more rigid and less responsive to biochemical and mechanical stimuli, thus impeding complexity generation in the branching epithelium of the developing kidney.

## Materials and methods

### Mice and kidney experiments

All experiments were approved by the Finnish Animal Care and Use Committee (license KEK16‐020) in compliance with all relevant ethical regulations regarding animal research. Experimental females were group‐housed in individually ventilated cages, while males were housed alone to avoid fighting. Mice were housed in a room where the temperature was kept at 23 ± 2°C with a 12‐h light/dark cycle (lights on at 6:00 a.m.) with free access to food and water. The design and genotyping of HoxB7CreGFP, *Mek1*‐floxed, *Mek2*‐null, beta‐catenin‐null, and beta‐catenin stabilized mice have been described [27, 43, 44, 82, 83] and were originally received through collaboration. Mice were of mixed genetic background with contributions from C57BL/6JOlaHsd (Janvier Laboratories, Le Genest‐Saint‐Isle, France). NMRI (Inotiv, Horst, the Netherlands) mice were used for MEK‐inhibition experiments.

For whole‐mount imaging, kidneys were dissected from embryos at E11.5 and E12.5, and shortly attached to Transwell filters (3 h, Transwell permeable supports, 0.4 μm polyester membrane, Corning Incorporated, Berlin, Germany) or cultured with inhibitor for 48 h. Kidneys were cultured in DMEM:F12 + GlutaMAX (Gibco, Paisley, Scotland) supplemented with 10% fetal bovine serum (FBS; Gibco) and penicillin–streptomycin (PS) (‘full DMEM’) under 5% CO_2_ at 37 °C [84]. MEK‐inhibition was achieved with 10–15 μm U0126 (Cell Signaling Technologies, Leiden, the Netherlands), Rho‐inhibition with 5 μm ROCKi (Y‐27632, STEMMCELL Technologies, Cologne, Germany) and adherens junction disruption with 80 μm EGTA (SIGMA, Espoo, Finland) in serum‐free media (DMEM + PS) and controlled by the addition of the same amount of dimethyl sulfoxide (DMSO) in serum‐free media to control kidney cultures.

Mechanical separation of the ureteric buds from E11.5–12.5 kidneys was carried out according to a published protocol [85]. Briefly, enzymatic collagenase treatment (4 mg·mL^−1^, 15 min at 37 °C) was followed by collagenase neutralization with 25 μ·mL^−1^ DNase I in DMEM/F12 + 10% FBS. Isolated UBs were cultured on fibronectin‐coated coverslips in DMEM/F12 + 10% FBS medium supplemented with GDNF (5 ng·mL^−1^), FGF2 (25 ng·mL^−1^), and HGF (50 ng·mL^−1^).

### Immunohistochemistry, electron microscopy, and imaging

The isolated UBs and UB‐derived cell line [86] (kindly donated by Dr Jonathan Barasch, Columbia university, NY, USA) were fixed with 4% paraformaldehyde (PFA) in PBS solution for 20 min at room temperature before being washed in PBS 3× 10 min. The permeabilization was performed with 0.1% Triton X‐100 + 50 mm glycine for 15 min, followed by PBS washes for 3× 10 min with gentle rocking. Cells were blocked for 30 min with 3% FBS in PBS, followed by primary antibody staining overnight in a cold room. Samples were washed in PBS for 3× 10 min before secondary antibody staining for 60 min. After 3× 10 min washes, nuclei were stained by Hoechst 33342 for 10 min. After follow‐up PBS washes, cover slips were mounted using the Epredia™ Immu‐Mount. The UB‐derived cell line is not authenticated and does not have an official name other than UB cells. All cells used in the experimentation were mycoplasma‐free.

For whole‐mount imaging, samples were fixed for 10 min in ice‐cold methanol and incubated with primary antibodies (goat E‐cadherin, R&D Systems, Minneapolis, MN, USA) and nuclear stain (Hoechst) diluted in PBS with 0.3% Triton X‐100 (PBST) at 4 °C twice o/n. Tissues were then washed for at least 3 × 30 min in PBST and incubated with secondary antibodies (anti‐goat Alexa Fluor 488, Jackson Immuno Research Laboratories, Cambrigde, UK) in PBST for 2 h at RT or o/n at 4 °C. Stained kidneys were washed at least 2 × 1 h + 1 × o/n in PBST and mounted with 99% glycerol (Sigma‐Aldrich). For paraffin sections, tissues were fixed with 4% PFA, embedded in paraffin using an automatic tissue processor, and cut to 5 μm thickness. Paraffin sections were rehydrated by xylene–alcohol series. Antigen retrieval was performed by simmering sections for 5–15 min in Tris–HCl EDTA buffer. Sections were blocked with 10% FBS for 1 h at RT prior to o/n incubation with primary antibodies at RT or 4 °C.

Glutaraldehyde (2%, Fluka) fixed E12.5 kidneys were processed for transmission electron microscopy (Tecnai G2 Spirit 120 kV TEM with Veleta and Quemesa CCD cameras, operated at 100 kV) as previously described [34]. Nuclei from 513 cells in 8 BC and 402 cells in 6 BI kidneys were manually segmented using the freehand tool in fiji (imagej). Measurements were taken and morphological characteristics were calculated using the ‘Shape descriptors’ function in fiji (imagej). *P* values were calculated by two‐tailed *t*‐test with unequal variance.

Confocal micrographs of whole‐mount and paraffin‐sectioned samples were taken with Leica SP8 except for E‐CAD quantification, which was carried out with Leica Thunder Imager Model Organism microscope. First, parallax correction for the z‐stack was applied, followed by Thunder processing using large volume computational clearance for glycerol mounting. Final images were created by applying extended depth of field for the processed z‐stack. Leica las‐x software was used to process the images. For whole‐mount imaging, z‐step was 0.333 μm and imaging depth varied from 80 to 100 μm, depending on the UB thickness.

### Machine learning‐based segmentation

Ilastik (https://www.ilastik.org) was used to perform segmentation of cell outlines based on the E‐cadherin staining as described previously [38]. Pixel classification in Ilastik works on a random forest algorithm that learns interactively from user annotations, including the selection of features for training and manual supervision of the training process. During this process, Ilastik learns to classify pixels into two different groups—membrane and non‐membrane. Ilastik outputs a probability map that can be used for further cell reconstruction.

### Extraction of spatial parameters and matlab analysis methods

For cell segmentation with ShapeMetrics, pixel classification from 3D confocal images was performed using ilastik [87], and segmentation was performed with a custom algorithm in matlab (MathWorks). In brief, Ilastik creates prediction maps for further processing of the image in matlab, followed by watershed segmentation and connection of possible gaps in cell borders. The segmented cells can be used to collate a single unbiased heatmap and divide it into subgroups according to selected cell features [38] In total, 11 BC and 3 BI UB tips were segmented (Tables 1 and 3).

The basic 3D reconstruction of UB tip cells, based on the probability maps from ilastik, extractions of geometric parameters, and clustergram analysis was done using the original version of the ShapeMetrics script (https://github.com/KerosuoLab/Shapemetrics), as described previously [38]. In ShapeMetrics, geometrical parameters are quantified from reconstructed cells. Additionally, a new script was written. This code sorts parameter values from highest to lowest and allows the selection of specific subgroups of cells for further characterization, as used here for the top 10% of cells. The geometric parameters we extracted are: volume, roundness, ellipticity, elongation, longest axis, minor axis, and intermediate axis. For visualization, we converted spatial data into numeric values for each parameter at a single‐cell resolution and performed principal component analysis and Uniform Manifold Approximation and Projection (UMAP) to two dimensions.

For both clustergram analysis and the 10%‐quantile method, merging of selected cells and original images was done in imagej. Merged multi‐pages were projected onto 2D images using the standard deviation algorithm in imagej. Distribution and whisker plots were made from the matrices generated in the ShapeMetrics script by using originpro. Basic column statistics including average, median, and skewness coefficient values were also calculated using originpro.

To statistically compare the differences in geometrical parameters among distinct UB morphologies (initial bud‐to‐ampulla; ampulla‐to‐asymmetric ampulla; asymmetric ampulla‐to‐T‐bud; T‐bud‐to‐initial bud) and BI epithelia, we performed a Kruskal–Wallis test, followed by a *post hoc* Conover‐Iman test for pairwise comparisons. These are non‐parametric, rank‐based comparisons without normal distribution requirement. Pairwise Levene's test was used to analyze variance between the geometries at different tip stages as well as across all tip morphologies and BI epithelia. Pairwise comparisons between geometries at different tip stages as well as across all tip morphologies and BI epithelial cells within shared volume ranges were performed by pairwise Mann–Whitney tests.

### Quantification of apical E‐CADHERIN in UB tips

The kidneys stained with E‐CAD were mounted in glycerol and imaged with Andor Dragonfly confocal with Andor Zyla 4.2 sCMO camera and Plan Apo VC 60× water objective. Imaging was carried out by running a z‐stack (0.15 μm per step) through the tip, and final images were deconvoluted in fusion 2.0 software (Oxford Instruments, Abingdon, UK). Maximum projections were made in Imaris using the Oblique Slicer option; mid‐section was created through the tips to visualize the apical membrane as much as possible. E‐CAD intensity profile was measured using the Measurement Points option in Imaris by selecting 20 random points along the apical membrane per each tip (control (DMSO) *n* = 4 tips/19 points, U0126 *n* = 4 tips/10 points, ROCKi *n* = 4 tips/16 points, EGTA *n* = 4 tips/10 points).

### Cell lines and transfections

The UB‐derived cell line [86] was cultured in DMEM with 10% FBS, 1× GlutaMAX, and 1× Normocin. UB‐derived cells were transfected with green fluorescent protein (GFP)‐tagged paxillin using Lipofectamine 3000 and co‐stained with E‐cadherin, endogenous paxillin, and overexpressed paxillin. The cell line was also transfected with GFP‐tagged E‐cadherin modified for improved surface expression [88]. These cells were co‐stained with E‐cadherin, endogenous paxillin, and overexpressed E‐cadherin.

### Traction force microscopy

Traction force microscopy of cell doublets was conducted as described previously [89, 90]. Micrographs were taken using a Zeiss Axiovert inverted microscope coupled to a CSUX1 spinning‐disc device (Yokogawa, Tokyo, Japan) in an environmental chamber at 37 °C and 5% CO_2_. In brief, polyacrylamide gels of 19.66 kPa elasticity were embedded with 0.2 μm 505/515 fluorescent beads (Life Technologies, Bleiswijk, the Netherlands) and coated with fibronectin (R&D Systems). UB‐derived cells were seeded at a density of 50 000 cells per gel to achieve cell doublet formation and incubated once o/n in full media and once more o/n with 15 μm U0126 or DMSO in serum‐free media prior to imaging. After all doublets were imaged (experimental), 10× trypsin (Gibco) was added, and cells were completely detached. Gel‐embedded fluorescent beads were imaged again in the absence of cells (reference). A custom Fourier‐Transform Traction Cytometry algorithm was used to calculate tractions based on bead displacement between ‘experimental’ and ‘reference’ images, described in detail previously [90].

For cell monolayers, a modified traction force microscopy approach was used [91, 92]. Briefly, cells were seeded upon fibronectin‐coated (R&D Systems) Softrac hydrogels (Matrigen, Irvine, CA, USA) with 25 kPa stiffness, embedded with 0.2 μm yellow‐green fluorescent microspheres in 35 mm dishes. The experiment was performed in three parts: first with a cell line derived from renal UB epithelium at 80% confluence, then again at 100% confluence, and third with primary UB epithelium excised from embryonic kidneys and allowed to delaminate onto the gels. In each part, for each cell monolayer, we obtained fluorescent microspheres images with and without cells present. From the image pair, we determined the cell‐exerted displacement field, and from the displacement field, the traction field using the method of Fourier‐Transform Traction Cytometry, modified to the case of cell monolayers. We report traction as the root mean square value within the field of view (yy) and averaged them across cell monolayers from each experimental part. Images were taken using 3i Marianas attached to a fully motorized Zeiss Axio Observer Z1 microscope at 37 °C and 5% CO_2_.

### On‐cell Western

Cells from the UB‐derived cell line were plated at a density of 12 500 cells per well in a black, clear‐bottom 96‐well plate (Perkin Elmer, Hägersten, Sweden). Prior to seeding, all wells were coated with 10 μg·mL^−1^ fibronectin (R&D Systems). Cells were cultured in DMEM:F12, 10% FBS, GlutaMAX, and normocin under 5% CO_2_ at 37 °C for 24–36 h, until they reached a confluence of 80–90%.

After reaching the desired confluence, cells in 48 wells were treated with 15 μm U0126 in DMEM/normocin. Cells in the other 48 wells were controlled by the addition of DMSO in DMEM/normocin. Cells were incubated with treatment for 24 h. Media containing U0126/DMSO was changed once during the incubation.

Cells in 60 wells were incubated with primary goat polyclonal anti‐mouse E‐cadherin (R&D Systems, Cat. No AF748, 1 : 200) in cold 5% FBS/PBS for 20 min on ice. Negative control cells (36 wells) were incubated only with cold 5% FBS/PBS. Cells were rinsed three times with cold PBS and fixed with 4% PFA for 10 min at room temperature. All wells were washed 3 × 5 min with PBS. After washing, blocking was performed for 15 min with Odyssey blocking buffer (PBS) (LI‐COR Biosciences, Bad Homburg, Germany, Cat. No 927‐40000) at room temperature followed by a 20 min incubation in the dark with IRDye 800CW donkey anti‐goat antibody (LI‐COR Biosciences, Cat. No 926‐32214, 1 : 1000) in 50% blocking buffer and 50% PBS/0.2% Tween‐20. Cells were co‐stained with nuclear stain: DRAQ5 (Thermo Fisher Scientific, Eindhoven, the Netherlands, Cat. No 62251, 1 : 7500) for normalization. Cells were then washed 3 × 10 min with PBS/0.1% Tween‐20. After washing, PBS was changed to wells and the plate was scanned with the LI‐COR Odyssey IR Imager (169 μm resolution, 3 focus offset, and 6 to 8 intensity). Data was analyzed using Excel (Microsoft).

The mean intensity value of negative controls was subtracted from the 800/700 intensity ratio. Significance of variability between conditions was determined using the Student's *t*‐test (two‐tailed, unpaired). Results with *P < 0.05, **P < 0.01, and ***P < 0.001 were considered significant. All experimental data are reported as means, and error bars represent experimental standard error (± standard deviation, SD).

### Functional gene enrichment analysis

Functional gene enrichment analysis of differentially expressed genes identified in BI UB epithelium (HoxB7Cre‐GFP;*Mek1*fl/fl;*Mek2* ^−/−^) [33] was performed using the ToppFun application from ToppGene Suite (https://toppgene.cchmc.org) [93]. GO enrichment of differentially expressed genes was performed with a false discovery rate of *P* < 0.05. GO biological processes shown in Fig. S5 were selected based on their possible relation to the observed phenotype in branching‐incompetent UBs.

## Conflict of interest

The authors declare no conflict of interest.

## Author contributions

KK, VI, and SK designed the experiments; SK conceptualized the project. KK, VI, HA, TZ, and JK conducted the experiments. DB provided beta‐catenin deficient kidney samples, and PC performed UMAP analyses. KK, VI, OJMM, and RK analyzed data. KK, VI, and SK wrote and edited the manuscript.

## Supporting information


**Table S1.** Individual ureteric bud tip quantification data in branching‐competent kidneys.

**Video S1.** Time‐lapse imaging of actin dynamics in the leading edge of primary UB epithelial cell culture set‐up.

## Data Availability

The data that support the findings of this study are available in Figs 1, 2, 3, 4, 5, 6, 7, 8, 9, 10, 11, 12, Tables 1, 2, 3, 4, and the Supporting Information of this article.
